# Supplementary figures and images for: Time-Resolved Imaging Reveals Heterogeneous Landscapes of Nanomolar Ca2+ in Neurons and Astroglia
Source: Neuron. 2015 Oct 21;88(2):277–88. doi: 10.1016/j.neuron.2015.09.043 (PMC4622934; doi:10.1016/j.neuron.2015.09.043)

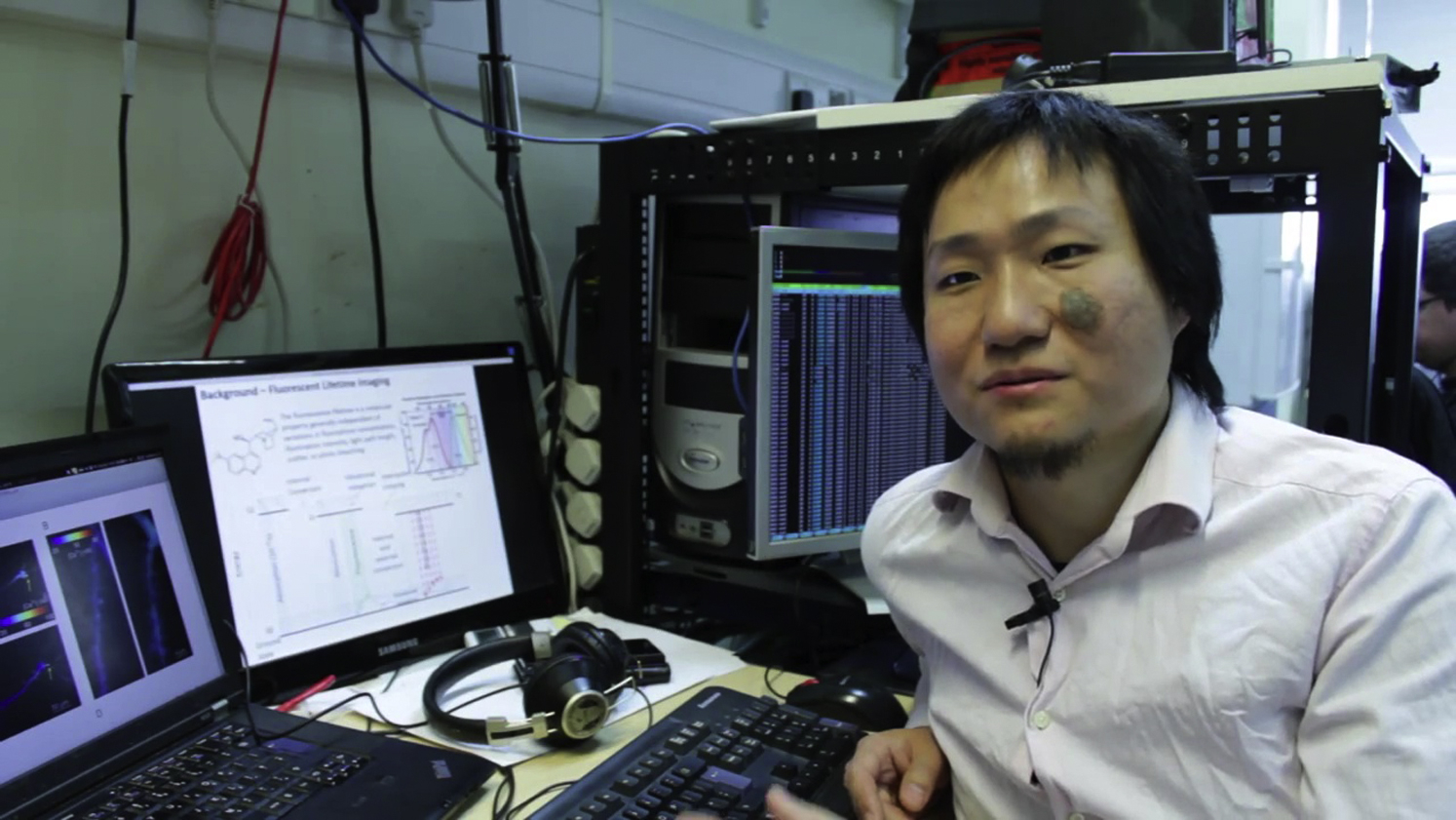

Supplement: Supplementary file 1 [file mmc4.jpg]

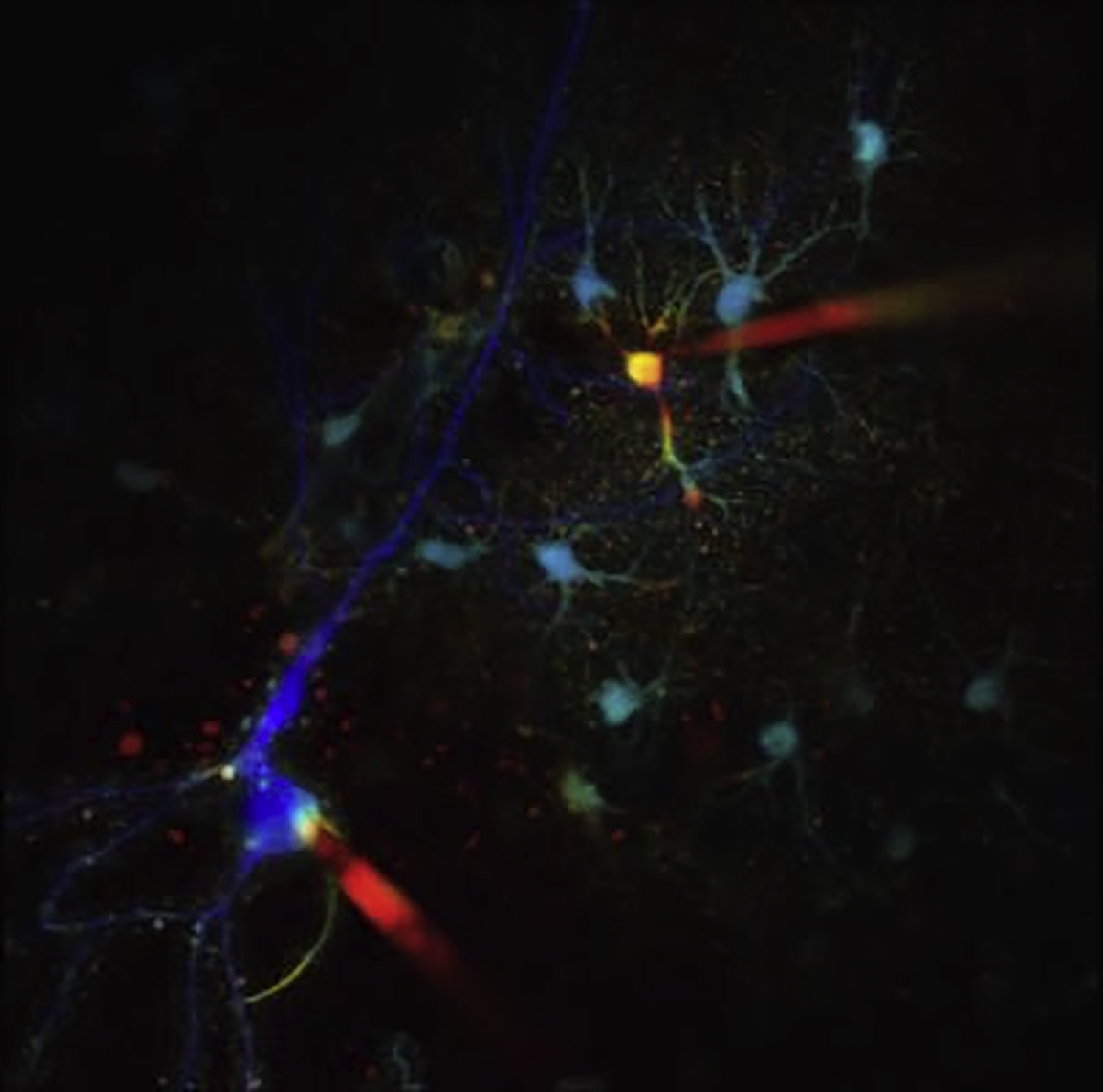

Supplement: Movie S1. Projection of OGB1-1, Related to Figure 3 [file mmc2.jpg]
