# Supplementary material for: Time-Resolved Imaging Reveals Heterogeneous Landscapes of Nanomolar Ca2+ in Neurons and Astroglia
Source: Neuron. 2015 Oct 21;88(2):277–88. doi: 10.1016/j.neuron.2015.09.043 (PMC4622934; doi:10.1016/j.neuron.2015.09.043)
Supplement: Document S1. Figures S1–S5 [file mmc1.pdf]

Neuron, Volume *88*

Supplemental Information

Time-Resolved Imaging Reveals Heterogeneous Landscapes of Nanomolar Ca^{2+} in Neurons and Astroglia

Kaiyu Zheng, Lucie Bard, James P. Reynolds, Claire King, Thomas P. Jensen, Alexander V. Gourine, and Dmitri A. Rusakov

SUPPLEMENTARY FIGURES

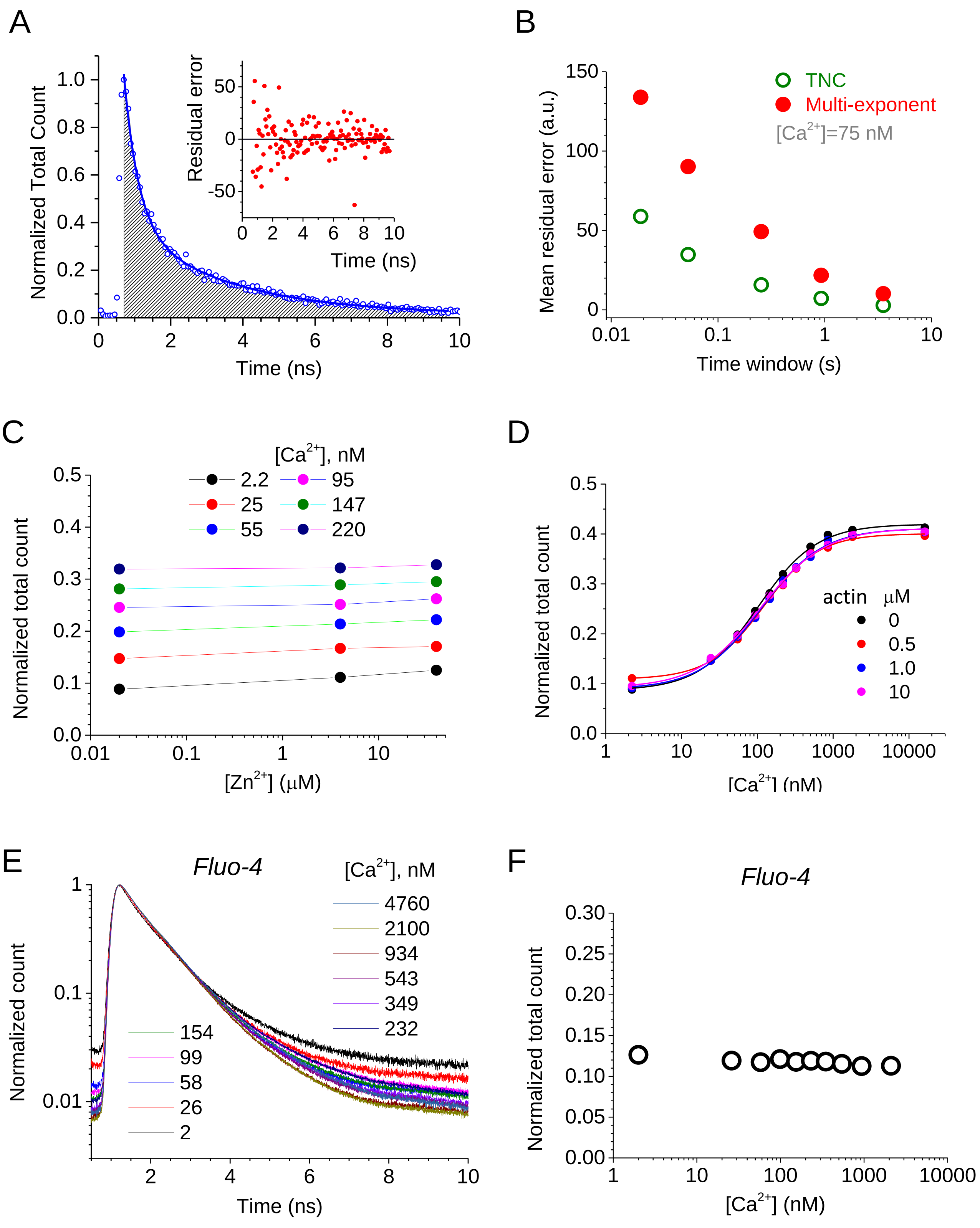

Figure S1 (Related to Fig. 1). Optimisation and concomitant sensitivity tests for the [Ca^{2+}]-sensitive OGB-1 fluorescence decay time course.

(A) Quantification of the OGB-1 fluorescence decay using Normalised Total Count (NTC) method *versus* double-exponent (DE) fitting method. Graph, example fluorescence decay trace (75 nM [Ca^{2+}] calibration solution); blue circles, raw photon count data; shaded area, integration area for NTC; blue curve, DE fitting (interval show by shade). Inset, plot of residuals (red dots) illustrating the fitting error for the DE method.

(B) Average error for [Ca^{2+}] estimation with FLIM (75 nM [Ca^{2+}] calibration solution) using the NTC (hollow green circles) or the DE (solid red circles) method with respect to different acquisition times (integration periods). As expected, increasing the acquisition time reduces the noise in the FLIM decay traces. Because of the higher sensitivity of the (non-linear) DE approximation to the experimental noise compared to the (linear) NTC approach, its overall error consistently exceeds that of the NTC method; a.u., arbitrary units.

(C) The sensitivity of OGB-1 fluorescence lifetime to [Ca^{2+}] at various free [Ca^{2+}] levels (using calibrated Ca^{2+} solutions, as indicated) is negligibly affected by [Zn^{2+}] in the 0.01-4 μM range.

(D) The sensitivity of OGB-1 fluorescence lifetime to [Ca^{2+}] is not affected by the presence of (a ubiquitous neuronal protein) actin over the range of 0-10 μM.

(E) The Fluo-4 fluorescence decay (photon count data) in calibrated solutions of concentration-clamped [Ca^{2+}], as indicated.

(F) Normalized total photon count ([Ca^{2+}] readout) for experiments in E shows little sensitivity of Fluo-4 to [Ca^{2+}].

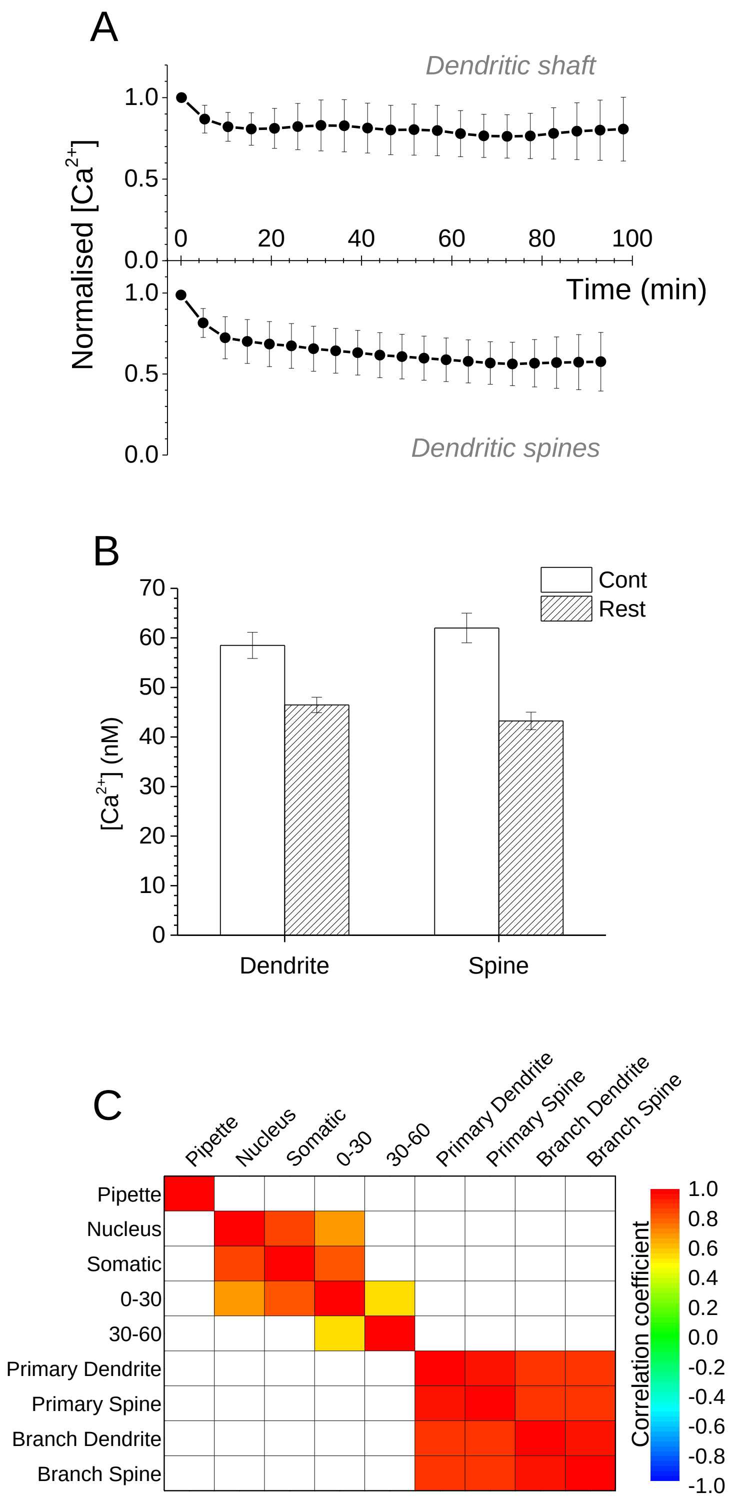

Figure S2 (Related to Fig. 2). Testing $[Ca^{2+}]$ in CA1 pyramidal cells readout at varied imaging conditions.

(A) Time course of average resting $[Ca^{2+}]$ (Normalized photon count for OGB-1 FLIM readout) after whole-cell break-in, in dendritic shafts and spines of CA1

pyramidal cells, as indicated. Dots (mean ± SEM), values averaged over multiple regions of interest throughout the dendritic tree in n = 3 cells.

(B) Average [Ca^{2+}] values (mean ± SEM) for two modes of image acquisition, continuous (Cont, n = 77) and with 3 second intervals between individual frames (Rest, n = 109 frames). Further increases in the inter-frame interval had no effect; one-cell example.

(C) Correlation coefficients for [Ca^{2+}] values across various cell compartments among cells in an experimental sample of n = 35, as indicated. Only statistically significant ($p < 0.05$) Pearson's correlation values are shown, empty table cells correspond to insignificant correlation.

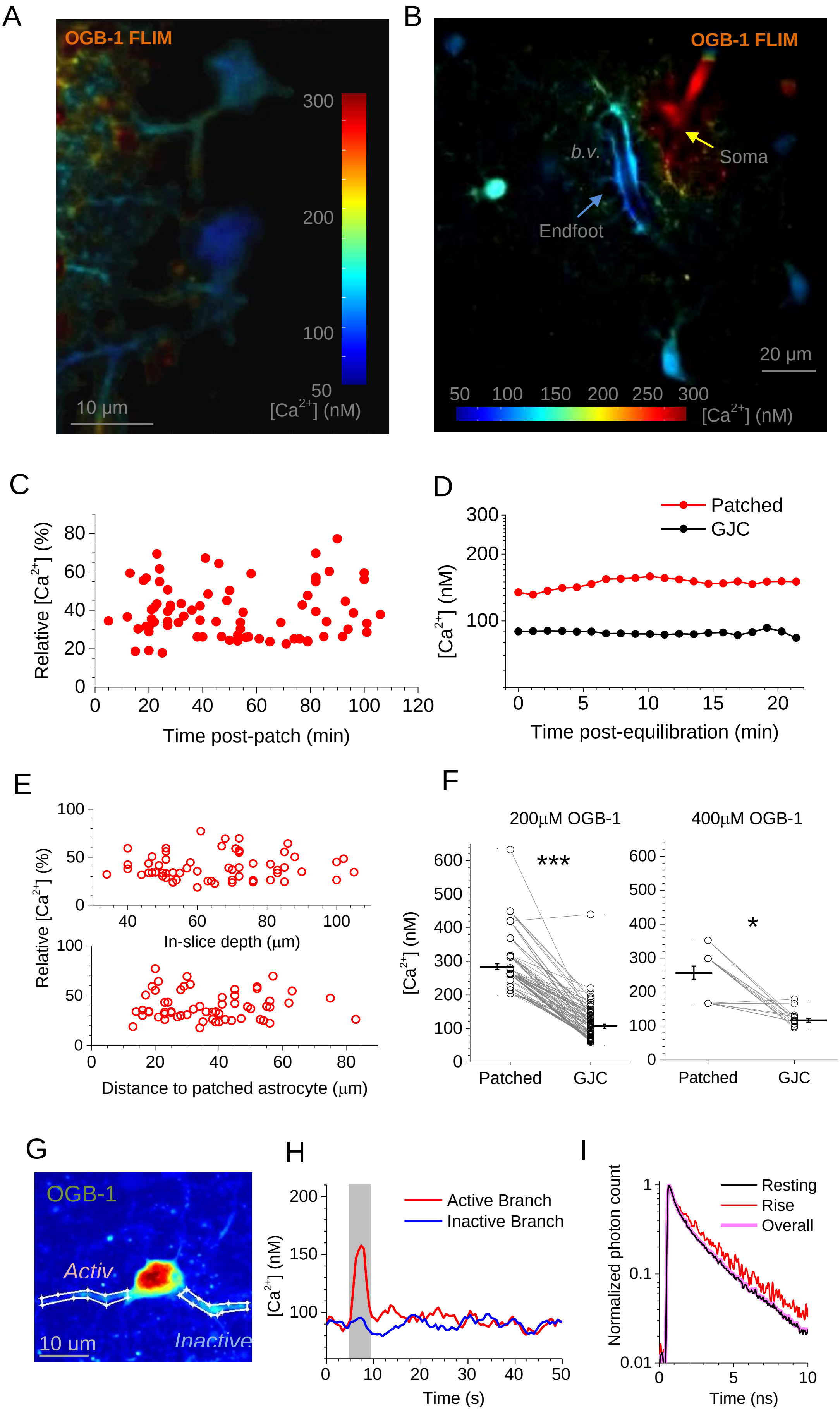
A
OGB-1 FLIM
300
200
100
50
[Ca2+] (nM)
10 µm
B
OGB-1 FLIM
b.v.
Soma
Endfoot
20 µm
50 100 150 200 250 300
[Ca2+] (nM)
C
Relative [Ca2+] (%)
Time post-patch (min)
D
Patched
GJC
[Ca2+] (nM)
Time post-equilibration (min)
E
Relative [Ca2+] (%)
In-slice depth (µm)
Distance to patched astrocyte (µm)
F
200µM OGB-1
400µM OGB-1
***
*
[Ca2+] (nM)
Patched
GJC
G
OGB-1
Activ
Inactive
10 um
H
Active Branch
Inactive Branch
[Ca2+] (nM)
Time (s)
I
Resting
Rise
Overall
Normalized photon count
Time (ns)

Figure S3 (related to Fig. 3). Monitoring basal [Ca^{2+}] in patched (whole-cell) and gap junction (GJ) -connected astroglia with OGB-1 FLIM readout.

(A) Examples of two GJ-connected astrocytes with false color-mapping for [Ca^{2+}] using OGB-1 NTC readout. A significant [Ca^{2+}] gradient between the somata and cell arbor peripheries can be seen, with resting [Ca^{2+}] values rising towards the periphery (in contrast to what is seen in a patched cell); see Fig. 3E for summary statistics.

(B) In a patched astrocyte (yellow arrow, patch pipette tip), the endfoot surrounding a blood vessel (*b.v.*, blue arrow) shows a 2-3 times lower [Ca^{2+}] than that near the soma; an example.

(C) Average [Ca^{2+}] in GJ-connected astrocytes does not depend on the time lapsed after cell patching, i.e. duration of dye diffusion through GJs (average for n = 78 cells).

(D) An example documenting the time course of average [Ca^{2+}] in one patched and one GJ-connected astrocyte (somatic area), as indicated, over the experiment duration.

(E) Average [Ca^{2+}] in GJ-connected astrocytes does not depend on the recording depth in slice (upper graph; dots, individual cell data; n = 62) or on the distance from the patched astrocyte (lower graph; n = 61); [Ca^{2+}] values shown relative to [Ca^{2+}] in the patched astrocyte soma.

(F) A two-fold increase in the loaded OGB-1 concentration has no effect on resting [Ca^{2+}], in either patched or GJ-connected astroglia, nor on the average [Ca^{2+}] difference between the two groups (somatic areas analyzed). Dots, individual cell data (n = 79 and n = 16 cells for 200 μM and 400 μM OGB-1, respectively); horizontal bars, mean ± SEM; *$p < 0.05$, ***$p < 0.001$.

(G) Example image (OGB-1 intensity, false color scale, planar projection) of an astrocyte with two delineated regions of interest in two (primary) cell branches, one of which (*Active*) did while the other (*Inactive*) did not generate a spontaneous [Ca^{2+}] rise during the recording period of 50 s.

(H) OGB-1 NTC-calibrated average [Ca^{2+}] time course in the two regions of interests depicted in G, as indicated. Note a similar magnitude of near-rest [Ca^{2+}] fluctuations

in both branches confirming a similar dynamic range of the two recordings. Grey shade, Ca^{2+} rise interval in the *Active* branch.

(I) Fluorescence (NTC) decay time course for OGB-1 FLIM in the *Active* branch recording trace (shown in G-H) sampled outside Ca^{2+} rise interval (Resting, black), inside the interval (Rise, red), and throughout the recording (Overall, magenta). Including the active [Ca^{2+}] rise event appears to have little effect on the overall readout value.

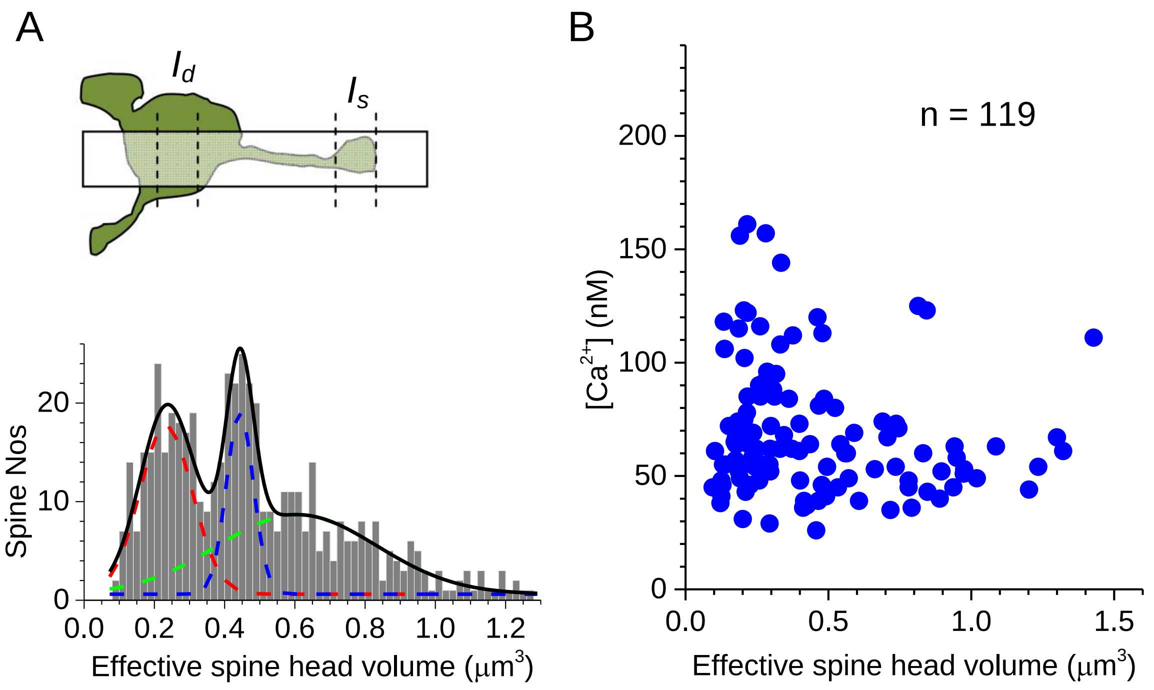

Figure S4 (Related to Fig. 4). The relationship between the size of dendritic spine heads and local resting [Ca^{2+}].

(A) Upper panel, diagram (vertical section of the dendritic fragment) depicting the spine head volume measurement which involves the ratio between the spine head fluorescence intensity (I_s) and that of the dendritic shaft in the same focal plane (I_d); white rectangle illustrates the characteristic focal two-photon excitation width: the volume scale was validated and adjusted *post hoc* using the average spine head size measurement (median width 519 nm) recently obtained in a spine population form the same hippocampal area using super-resolution (STED) microscopy (Tonnesen et al., 2014). Histogram, frequency distribution of spine head volumes (n = 536) suggests two or three spine subgroups with respect to head size. Dotted lines, three Gaussian distributions; solid line, the sum (histogram best fit).

(B) In proximal (first order) apical dendrites of CA1 pyramidal cells the spine head volume is not correlated with the intra-head [Ca^{2+}] measured with OGB-1 FLIM readout. Dots, individual dendritic spine recordings.

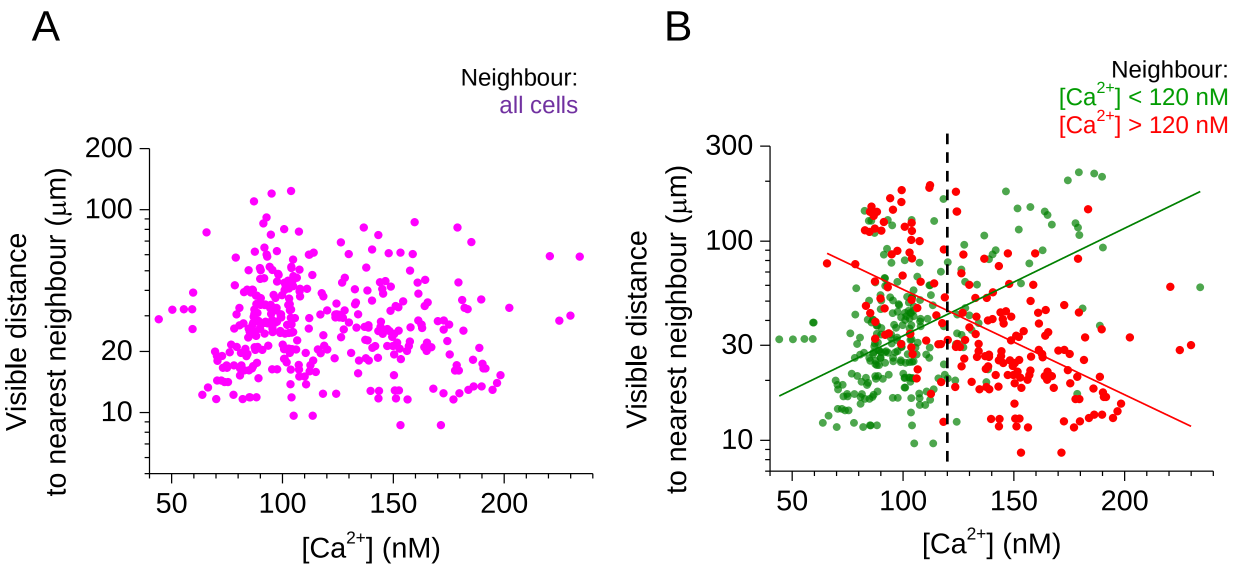

Figure S5 (Related to Fig. 5). Nearest-neighbour distances and somatic $[Ca^{2+}]$ in cortex astroglia *in vivo*.

(A) Distance to the nearest neighbor from individual, randomly sampled astrocytes plotted against $[Ca^{2+}]$ in the sampled astrocyte (n = 343 cells).

(B) Distance to the astrocyte's nearest neighbor plotted against astrocyte $[Ca^{2+}]$, with the nearest-neighbor astrocytes marked in accordance with one of their two somatic $[Ca^{2+}]$ sub-group (see Fig. 5E), as indicated: green, $[Ca^{2+}]$ < 120 nM (n = 301); red, $[Ca^{2+}]$ > 120 nM (n = 327), with the 120 nM boundary depicted by vertical dotted line. The plot indicates that the distance from any lower-$[Ca^{2+}]$ cell (green) to its nearest neighbor increases with higher $[Ca^{2+}]$ in that neighbor (green line, linear regression at $F = 88.7$, $p < 0.001$); conversely, the distance from any higher-$[Ca^{2+}]$ cell (red) to its nearest neighbor increase with lower $[Ca^{2+}]$ in that neighbor (red line, linear regression at $F = 81.8$, $p < 0.001$). In other words, astrocytes from the same $[Ca^{2+}]$ group tend to occur substantially closer to one another compared to those sampled from the two different groups.
