# Supplementary material for: Time-Resolved Imaging Reveals Heterogeneous Landscapes of Nanomolar Ca2+ in Neurons and Astroglia
Source: Neuron. 2015 Oct 21;88(2):277–88. doi: 10.1016/j.neuron.2015.09.043 (PMC4622934; doi:10.1016/j.neuron.2015.09.043)
Supplement: Document S2. Article plus Supplemental Information [file mmc3.pdf]

NeuroResource

Neuron

Time-Resolved Imaging Reveals Heterogeneous Landscapes of Nanomolar Ca^{2+} in Neurons and Astroglia

Highlights

- OGB-1 lifetime readout is specific to low [Ca^{2+}] and enables 100 Hz acquisition rate
- Neurons and astrocytes maintain uneven landscapes of resting [Ca^{2+}]
- Resting [Ca^{2+}] patterns in neurons and astroglia are age and activity dependent
- Astroglia in situ split into two sub-groups with respect to their resting [Ca^{2+}]

Authors

Kaiyu Zheng, Lucie Bard, James P. Reynolds, Claire King, Thomas P. Jensen, Alexander V. Gourine, Dmitri A. Rusakov

Correspondence

d.rusakov@ucl.ac.uk (D.A.R.),
k.zheng@ucl.ac.uk (K.Z.)

In Brief

Zheng et al. advance a fluorescence imaging technique enabling the monitoring of very low (nanomolar) calcium concentrations inside brain cells in situ with high resolution. The method uncovers rich information content of resting calcium landscapes inside neurons and astroglia.

Zheng et al., 2015, Neuron *88*, 277–288
October 21, 2015 ©2015 The Authors
http://dx.doi.org/10.1016/j.neuron.2015.09.043

CellPress

Time-Resolved Imaging Reveals Heterogeneous Landscapes of Nanomolar Ca^{2+} in Neurons and Astroglia

Kaiyu Zheng,[1,3,*] Lucie Bard,[1,3] James P. Reynolds,[1] Claire King,[1] Thomas P. Jensen,[1] Alexander V. Gourine,[2] and Dmitri A. Rusakov[1,*]
[1]UCL Institute of Neurology, University College London, Queen Square, London WC1N 3BG, UK
[2]Department of Neuroscience, Physiology and Pharmacology, University College London, Gower Street, London WC1E 6BT, UK
[3]Co-first author
*Correspondence: d.rusakov@ucl.ac.uk (D.A.R.), k.zheng@ucl.ac.uk (K.Z.)
http://dx.doi.org/10.1016/j.neuron.2015.09.043
This is an open access article under the CC BY license (http://creativecommons.org/licenses/by/4.0/).

SUMMARY

Maintaining low intracellular calcium is essential to the functioning of brain cells, yet the phenomenology and mechanisms involved remain an enigma. We have advanced a two-photon excitation time-resolved imaging technique, which exploits high sensitivity of the OGB-1 fluorescence lifetime to nanomolar Ca^{2+} concentration ($[Ca^{2+}]$) and enables a high data acquisition rate in situ. The $[Ca^{2+}]$ readout is not affected by dye concentration, light scattering, photobleaching, micro-viscosity, temperature, or the main known concomitants of cellular activity. In quiescent tissue, standard whole-cell configuration has little effect on resting $[Ca^{2+}]$ inside neuronal dendrites or inside astroglia dye-filled via gap junctions. Mapping basal $[Ca^{2+}]$ in neurons and astrocytes with submicron resolution unveils heterogeneous concentration landscapes that depend on age and preceding activity. The rich information content represented by such landscapes in acute slices and in vivo promises to unveil the hitherto unexplored, potentially fundamental aspects of brain cell physiology.

INTRODUCTION

Neural communication in the brain relies on the homeostasis and dynamics of intracellular Ca^{2+}. In neurons, release of neurotransmitters is controlled by presynaptic Ca^{2+} entry (Katz and Miledi, 1968), whereas memory trace formation depends on postsynaptic Ca^{2+} transients in dendritic spines (Lisman, 1989; Nishiyama et al., 2000). Subtle alterations in basal $[Ca^{2+}]$ play a key role in nerve cell signal transduction cascades (Ross, 2012), also correlating with aging (Toescu and Verkhratsky, 2007). More recently, it has emerged that brain astroglia contribute to signal transfer in local neural circuits (Halassa and Haydon, 2010; Perea et al., 2009; Volterra and Meldolesi, 2005). Electrically passive astrocytes handle physiological messages primarily through intracellular Ca^{2+} waves that show multiple spatiotemporal modalities in situ and in vivo (Di Castro et al., 2011; Gee et al., 2014; Shigetomi et al., 2013a), including slow changes in basal $[Ca^{2+}]$ (Shigetomi et al., 2012). Accurate $[Ca^{2+}]$ monitoring in neurons and glia therefore remains crucial to our understanding of brain function.

Historically, ratiometric Ca^{2+} indicators applied in cell cultures have provided accurate measurements of intracellular $[Ca^{2+}]$ and its slow changes (Grynkiewicz et al., 1985; Tsien, 1989). However, the use of such indicators in situ has been limited because light absorption and scattering in organized tissue are strongly wavelength dependent and furthermore could vary with activity or development (Grienberger and Konnerth, 2012; Oheim et al., 2001). These factors introduce poorly controlled errors to the ratiometric readout, which relies on the pre-determined signal ratios at different wavelengths. Instead, $[Ca^{2+}]$ monitoring in situ has commonly been carried out using fluorescence intensity-based measures (Grienberger and Konnerth, 2012; Maravall et al., 2000). While providing robust readout for rapid $[Ca^{2+}]$ dynamics, the accuracy of the latter approach for low basal $[Ca^{2+}]$ (below 100 nM) is limited by the errors pertinent to the low signal-to-noise ratio, fluctuations in the dye concentration, focus drift, or photobleaching (Oertner et al., 2002; Scott and Rusakov, 2006). Evaluating resting $[Ca^{2+}]$ and its intracellular distribution in situ has thus remained a challenge, yet this knowledge appears essential for deciphering Ca^{2+} signals in various physiological scenarios.

In contrast to the common intensity-based measurements, fluorescence lifetime imaging (FLIM) exploits the fact that photon emission is a stochastic event occurring for up to several nanoseconds post-excitation, with a progressively declining probability. By registering a large number of emission events, FLIM thus restores the fluorescence signal decay time course (see below). This decay could be highly sensitive to molecular microenvironment; FLIM techniques have been emerging that use specific indicators to monitor nanometer-scale molecular interactions in live cells (Koldenkova and Nagai, 2013; Ueda et al., 2013).

Because the fluorescence decay does not depend on how many dye molecules are imaged or how many photons are counted (these numbers will instead affect the measurement error), FLIM readout is inherently insensitive to the emission intensity or dye concentration. Consequently, it is also insensitive to

focus fluctuations or light scattering, which all represent the key factors hampering fluorescence imaging in situ. It has also been found that the fluorescence lifetime of some commonly used Ca^{2+} dyes, such as Oregon Green BAPTA-1 (OGB-1), is sensitive to free Ca^{2+} in the nanomolar range (Wilms and Eilers, 2007; Wilms et al., 2006). This feature of OGB-1 has led to a successful evaluation of bulk changes in astroglial [Ca^{2+}] in a mouse model of Alzheimer's disease (Kuchibhotla et al., 2009). However, because FLIM readout can be sensitive to other variables of the intracellular milieu (Baylor and Hollingworth, 2011), such as pH (Battisti et al., 2012), temperature (Tsuji et al., 2013), or medium viscosity (Wu et al., 2013), validating the method accordingly has remained an important issue.

Here we show that the Ca^{2+}-dependent lifetime of OGB-1 excited in two-photon mode is insensitive to physiological changes in pH, [Mg^{2+}], [Zn^{2+}], a ubiquitous neural protein (actin), temperature, or micro-viscosity. We implement a FLIM photon-counting procedure that boosts the readout stability, enabling a FLIM-mode image acquisition in situ at 100 Hz. We adapt this technique for high-sensitivity, high-resolution [Ca^{2+}] monitoring in neurons and astroglia in acute brain slices and in vivo. Our case study unveils some previously unattainable, striking features of the intracellular Ca^{2+} homeostasis in the brain, suggesting a strong gnostic potential for the present technique.

RESULTS

OGB-1 Lifetime Integrated Photon Counts Robustly Report Nanomolar [Ca^{2+}] in Varied Microenvironments

The in-house FLIM system used either a BioRad Radiance single-scanhead, or an Olympus FluoView dual-scanhead microscope (Figure 1A; Experimental Procedures). The FLIM duty cycle was driven by an 80 MHz infrared pulsed laser (SpectraPhysics Newport MaiTai). First, we calibrated OGB-1 FLIM readouts (at λ_x^{2P} = 800 nm) using standard solutions of buffered [Ca^{2+}] (Experimental Procedures). The outcome was readily consistent with previous reports (Wilms and Eilers, 2007; Wilms et al., 2006), displaying high FLIM readout sensitivity in the 10–200 nM [Ca^{2+}] range (Figure 1B). With no live tissue, the length of laser exposure in these tests was virtually unrestricted; this enabled large numbers of photon counts to follow the OGB-1 fluorescence decay with the required accuracy. In live-cell imaging, however, laser exposure is a major limiting factor; the maximal readout rate will be constrained by the minimal number of photon counts required to estimate [Ca^{2+}] from its FLIM calibration data (Figure 1B).

It was important therefore to develop a procedure that minimizes photon counts required to evaluate the OGB-1 fluorescence lifetime. We asked how the classical approach, which in the case of OGB-1 involves bi-exponential approximation of the fluorescence decay (suggested by the underlying physics) (Kuchibhotla et al., 2009; Wilms et al., 2006), compares with other estimators on the same datasets. Our systematic comparisons have revealed that direct integration of photon counts during a 9 ns window post-peak (termed here normalized total count, NTC; Figure S1A) produces a [Ca^{2+}] measurement error that is several times smaller than the error pertinent to the bi-exponential approximation (Figure S1B). Thus, NTC should substantially reduce the laser exposure time to obtain a required signal-to-noise ratio for [Ca^{2+}] monitoring, compared to a traditional method. Reassuringly, the single-value NTC estimator provided a calibration function for [Ca^{2+}] with an excellent sigmoid fit (Figure 1C).

Equipped with this measure, we next tested whether the FLIM [Ca^{2+}] readout was affected by the common micro-environment factors in the intracellular milieu. First, the tests showed excellent stability for [Ca^{2+}] evaluation over the wide range of pH (Figure 1D) or [Mg^{2+}] values (Figure 1E, upper panel), the two concomitants most likely to be affected by cellular activity. The FLIM readout was also unaffected by the length of laser exposure (Figure 1E, lower panel), thus ruling out the effects pertinent to photobleaching. Similarly, the NTC measure was not influenced by the temperature (19°C–36°C range, Figure 1F), by changes in the medium viscosity (altered by adding 70 kDa dextran; Figure 1G), or by [Zn^{2+}] fluctuations (Figure S1C). Some Ca^{2+} indicators, such as indo-1 and fura-2, were reported to show sensitivity in their Ca^{2+}-binding or spectral properties (although apparently not in the fluorescence lifetime) to ubiquitous cytoplasmic proteins in the muscle (Baker et al., 1994; Baylor and Hollingworth, 2011; Konishi et al., 1988). We found that adding the omnipresent neuronal protein actin to the calibration medium, in a range of plausible concentrations, had no effect on the [Ca^{2+}] FLIM measure (Figure S1D). Taken together, these tests appear to meet, or probably exceed, the non-specificity requirements expected from commonly used Ca^{2+} indicators. We thus deemed our OGB-1 FLIM readout (NTC) suitable for monitoring intracellular [Ca^{2+}] in situ. Interestingly, [Ca^{2+}] sensitivity of the OGB-1 fluorescence decay is not universal among Ca^{2+} dyes: the NTC readout of Fluo-4 was insensitive to [Ca^{2+}] (Figures S1E and S1F).

Mapping Resting [Ca^{2+}] in Individual Neurons in Acute Slices and In Vivo

To validate our approach for in situ [Ca^{2+}] measurements, we explored a well-established preparation of acute hippocampal slices (from P21–P24 rats, unless indicated otherwise; Experimental Procedures). We held a CA1 pyramidal neuron in whole-cell configuration (loaded with 200 μM OGB-1; quiescent slice conditions), as detailed previously (Sylantyev et al., 2013), and carried out pixel-by-pixel [Ca^{2+}] mapping in image stacks (Figure 2A; typical frame recording time 60–120 s, laser power < 8 mW under the objective). It normally took ~20 min after break-in for [Ca^{2+}] to stabilize in dendrites (Figure 2B) and ~30 min in dendritic spines (Figure S2B). We also established an optimal regime of collecting such images in FLIM mode. Resting [Ca^{2+}] remained stable at inter-frame intervals of >3 s, whereas during continuous frame acquisition it was slightly elevated (Figure S2B); the latter was consistent with residual photo-damage (Koester et al., 1999).

Because FLIM readout does not depend on the dye concentration, it should provide an unbiased measure for [Ca^{2+}] fluctuations pertinent to pipette dialysis and exogenous Ca^{2+} buffering. We therefore mapped [Ca^{2+}] at different distances from the somatic patch pipette (Figure 2A; [Ca^{2+}] remained at 200–300 nM inside the pipette tip, likely because of exposure to the bath medium prior to patching). Strikingly, in CA1

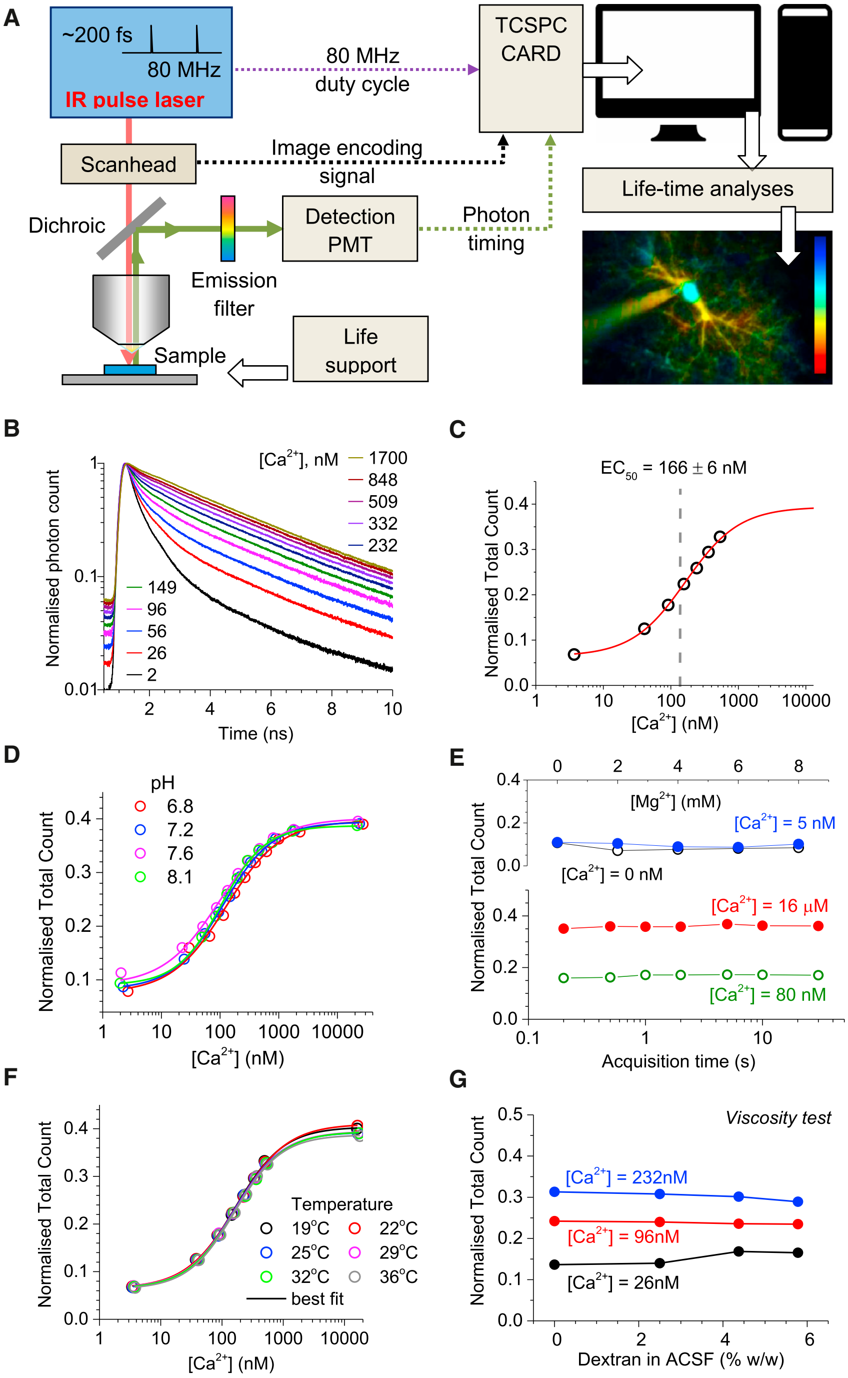

Figure 1. Oregon Green BAPTA-1 FLIM Readout Calibration for [Ca^{2+}]

(A) Diagram of the experimental imaging setup (main components) for in situ FLIM measurement and analysis (see Experimental Procedures for details).

(B) Normalized fluorescent lifetime decay curves of Oregon Green BAPTA-1 at various levels of clamped [Ca^{2+}] (calibrated solutions finely adjusted to include intracellular solution ingredients; Experimental Procedures). FLIM traces are normalized to their peak values (at ~1.3 ns post-pulse); (pH = 7.2) [Mg^{2+}] = 4 mM.

(C) The FLIM estimator, normalized total count, fitted with sigmoid type function (0.393 + (0.064 – 0.393) / (1 + (× / 165.6)$^{1.096}$); χ^2 = 1.33 × 10^{-5}, R^2 = 0.999).

(D) The OGB-1 FLIM readout is insensitive to pH over its plausible physiological range and throughout [Ca^{2+}] values, as indicated. Other notations are as in (C).

(E) FLIM estimator is insensitive to [Mg^{2+}] (upper graph) over the physiological range at near-zero [Ca^{2+}] (hollow black) and 5 nM (solid blue), as indicated, and to laser exposure at both high (solid red, 16 μM) and low (hollow green, ~80 nM) [Ca^{2+}] (lower graph).

(F) FLIM readout is insensitive to temperature over its physiological range and throughout [Ca^{2+}], as indicated. Other notations are as in (C).

(G) FLIM estimator is largely insensitive to viscosity at physiological ranges. 70 kDa dextran was added to ACSF, as indicated (see Zheng et al., 2014 for the corresponding viscosity measurements in vitro and in situ).

pyramidal neurons [Ca^{2+}] dropped to 57 ± 8 nM almost immediately outside the pipette tip (Figures 2A and 2C). The average [Ca^{2+}] remained low throughout the apical dendrites (n = 35 pyramidal cells, Figures 2A and 2C; note a slightly higher level in basal dendritic branches) over the recording time (30–120 min). Furthermore, [Ca^{2+}] values in individual dendritic compartments were well correlated among different cells (i.e., they were predictable rather than varied randomly) but not correlated with the residual patch-pipette [Ca^{2+}] (Figure S2C).

These observations indicate that principal neurons in situ possess a robust molecular machinery to maintain low [Ca^{2+}] in whole-cell mode. However, Ca^{2+} buffering indicators, combined with continuous patch-pipette dialysis, could still alter the endogenous resting [Ca^{2+}]. We therefore compared [Ca^{2+}] NTC readout for two OGB-1 concentrations (200 and 400 μM) and also in conditions of OGB-1 single-cell bolus-patch loading (whole-cell dialysis lasting only 2–3 min; Figure 2D; Experimental Procedures). The outcome showed no significant difference among tested OGB-1 loading protocols across the dendritic compartments (Figure 2F; the data suggest a slight trend toward lower [Ca^{2+}] at the higher OGB-1).

Finally, we implemented our FLIM measurement approach in vivo using local delivery of cell-permeable OGB-1 AM by microinjection via a pressurized pipette ("multiple-cell bolus loading"). In this protocol, the dye is taken up and subsequently hydrolyzed inside cells (Garaschuk et al., 2006; Experimental Procedures). Basal [Ca^{2+}] measured in readily identifiable somata of cortical neurons (n = 223 cells, layer II–III of somatosensory cortex; Figure 2E and Figure 3D) was on average higher than that in acute slices (mean ± SD; 82.4 ± 15.8 nM); this was

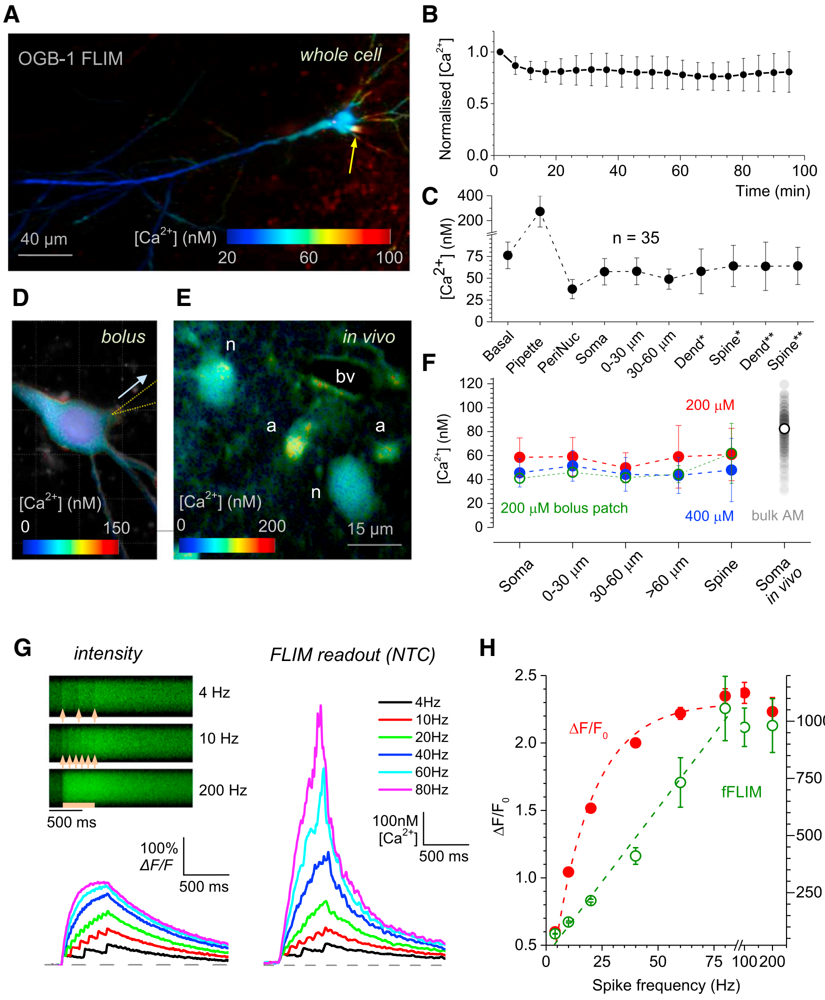

Figure 2. Mapping [Ca^{2+}] in CA1 Pyramidal Cells Using Two-Photon Excitation OGB-1 FLIM Readout

(A) Example of CA1 pyramidal cell mapped for the resting [Ca^{2+}] landscape in the soma, apical and basal dendrites, at the cell-wide map resolution (3D FLIM-coded image stack average shown); yellow arrow, patch pipette tip; free [Ca^{2+}] color coded, as indicated; brightness represents fluorescence intensity.

(B) Resting [Ca^{2+}] in secondary apical dendrites of CA1 pyramidal cells remains stable post break-in; dots (mean ± SEM), values averaged over visible fragments throughout the dendritic tree (n = 3 cells).

(C) Average resting [Ca^{2+}] (dots, mean ± SEM) measured in CA1 pyramidal cell compartments (n = 35 cells), at different distances from the patched soma, as indicated (<30 µm for basal dendrites); 200 µM OGB-1; "PeriNuc" denotes the somatic region associated with the visible cell nucleus; inclusion of the nearby cytoplasm is possible; * and ** denote local (~1 micron ROI) readouts from dendritic stems and spine heads in primary and secondary apical dendrites, respectively.

(D) Example of single-cell bolus-loaded CA1 pyramidal neuron mapped for resting [Ca^{2+}] in somatic proximity; arrow indicates withdrawal path of the patch pipette (dotted lines; pipette routinely withdrawn after 2–3 min in whole cell).

(E) Fragment of cortical neuropil in vivo FLIM-mapped for [Ca^{2+}], as indicated (single optical section); images depict neuronal (n) and astroglial (a) somata and a blood vessel (bv) section (see Figures 3C and 3D for further illustration and SR101-based astroglia selection).

(F) Average basal [Ca^{2+}] (dots, mean ± SEM) summarized for main cell compartments: at two concentrations of OGB-1 whole-cell loaded in acute slices (red, 200 µM, n = 19–44 depending on cell compartment; blue, 400 µM, n = 10–13), with single-cell bolus loading in slices (green, n = 11), and in cortical pyramidal cells in vivo (n = 223 neurons, somatosensory cortex 100–300 µm depth) loaded through OGB-1 AM puff injection, as indicated (gray; empty circle, mean).

(G) Comparison between intensity and OGB-1 NTC readout for Ca^{2+} entry in an apical dendrite in response to back-propagating spikes evoked at the soma at different frequencies, as indicated. Inset: example line scans depicting fluorescence intensity response for selected trials; traces, standard *ΔF/F* intensity readout (left) and the NTC readout calibrated for [Ca^{2+}] (right).

(H) Summary of experiments depicted in (G) (n = 5), as indicated; note saturation of the OGB-1 *ΔF/F* readout at ~1 µM [Ca^{2+}] and a linear relationship between fast FLIM (fFLIM) NTC readout and spike frequency, up to the saturation level.

most likely due to intermittent network activity, which is absent in quiescent slices. Taken together, these observations suggest that the active mechanisms of Ca^{2+} homeostasis in neurons (see Discussion) can cope with variable amounts of Ca^{2+} -buffering indicators commonly used in Ca^{2+} imaging studies.

Monitoring Rapid Ca^{2+} Dynamics in Neurons Using FLIM of OGB-1

Photon counting in FLIM requires hundreds of excitation cycles for every imaged pixel, and therefore it takes time to generate a detailed [Ca^{2+}] map (e.g., Figure 2A; 30–120 s for a single-section image). To enable registration of rapid [Ca^{2+}] changes evoked by neuronal activity, we therefore developed a procedure to record FLIM data (NTC readout) in a rapid imaging sequence, with 10 ms temporal resolution (Experimental Procedures). To validate the method in a typical imaging experiment, we recorded [Ca^{2+}] signals in neuronal dendrites following a 500 ms train of back propagating spikes evoked at frequencies between 4 and 200 Hz and used this dataset to compare the common intensity readout *ΔF/F* with the calibrated NTC readout (Figure 2G). As expected, *ΔF/F* response was highly sub-linear due to dye saturation (Figure 2G, left panel; Figure 2H). More importantly, translating these *ΔF/F* data into the [Ca^{2+}] time course would require detailed kinetic modeling and additional experimental controls (Ermolyuk et al., 2012; Rusakov, 2015). In contrast, NTC readout directly provided [Ca^{2+}] values and was linearly related to the spike frequency (Figure 2G, right) for up to 80 Hz firing rate (near-complete saturation; Figure 2H). The linear relationship between [Ca^{2+}] readout and spike frequency is an important feature because it could be critical for accurate registration of spiking with Ca^{2+} indicators in vivo (Grienberger and Konnerth, 2012).

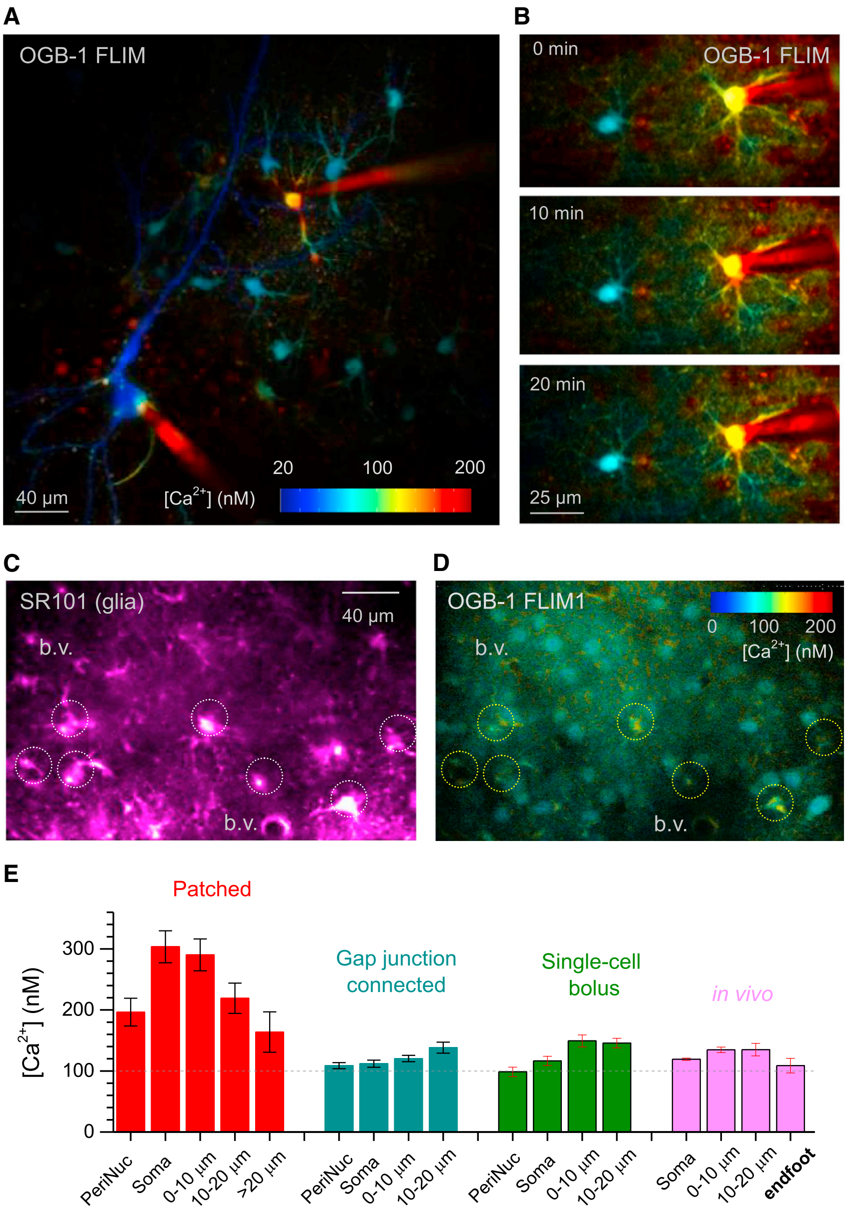

Figure 3. Mapping $[Ca^{2+}]$ in CA1 Astrocytes Using Two-Photon Excitation OGB-1 NTC Readout

(A) Landscapes of resting $[Ca^{2+}]$ obtained using FLIM for whole-cell OGB-1-loaded CA1 pyramid and astroglia (two pipette tips can be seen), including gap junction (GJ)-connected astrocytes; planar projection (see Movie S1 for 3D animation); false color scale is truncated at 200 nM to expand its dynamic range for lower $[Ca^{2+}]$.

(B) $[Ca^{2+}]$ map for a patched (pipette tip seen) and a GJ-connected astrocyte (left), taken at three 10 min intervals. Note sustained $[Ca^{2+}]$ gradients between and within cells; color-coded scale in (A) applies.

(C and D) In vivo $[Ca^{2+}]$ FLIM mapping of astroglia and pyramidal neurons (somatosensory cortex, layers II and III) seen in the SR101 channel highlighting astroglia (C) and in the OGB-1 FLIM channel (D, false color scale); dotted circles indicate several prominent astrocytes; b.v., blood vessel (sections) surrounded by the astrocyte endfoot process.

(E) Average $[Ca^{2+}]$ levels (mean ± SEM) at different distances from the soma of a patched astrocyte (red, n = 17 cells) in GJ-connected neighboring astroglia (cyan, n = 79); single-cell bolus loaded (green, n = 5 cells at 400 µM and n = 6 cells at 800 µM, combined), and in the astroglial somata and proximal processes in vivo (magenta, n = 357, 66, 9, and 17 for soma, 0–10, 10–20 µm areas, and astrocyte endfoot, respectively).

Measuring Resting $[Ca^{2+}]$ in Astroglia

To directly compare intracellular $[Ca^{2+}]$ between neurons and astroglia, we patched and loaded with OGB-1 the two cell types in close proximity and FLIM-mapped the area for $[Ca^{2+}]$ (Figure 3A; see Movie S1 for 3D animation). This experiment revealed several prominent phenomena. First, resting $[Ca^{2+}]$ in principal neurons was substantially lower than in neighboring astroglia. Second, the patched astrocyte showed elevated $[Ca^{2+}]$ compared to its neighbors filled with OGB-1 through the astrocyte-astrocyte gap junctions (GJs; Figure 3A), which are permeable to small (<1.5 kDa) indicators (Giaume et al., 2010). Third, these GJ-connected astrocytes appeared to maintain radial $[Ca^{2+}]$ gradients, with the concentration increasing toward the cell periphery (Figure 3B; Figure S3A). Conversely, in the patched cell $[Ca^{2+}]$ decreased toward the cell periphery (Figure 3B; Figure S3B).

Reassuringly, these observations were fully recapitulated in vivo: basal $[Ca^{2+}]$ in neurons was consistently lower than that in astroglia (compare Figures 2E with 3C and 3D). The $[Ca^{2+}]$ values and soma-periphery gradients were therefore being consistent throughout GC-connected, bolus-loaded, or intact in vivo astrocytes (Figure 3E). Thus, in astrocytes held in whole cell, $[Ca^{2+}]$ was elevated toward the soma, whereas in GJ-connected cells it was generally lower while showing the opposite, somatofugal elevation (Figure 3E). The latter trend was somewhat reversed in the endfoot processes in vivo (Figure 3E), which appear to control local vasculature in a Ca^{2+}-dependent fashion (Otsu et al., 2015).

Throughout imaged astroglia, basal intracellular $[Ca^{2+}]$, once equilibrated (10–15 min post-patch), remained remarkably stable, both across the cell population (Figure S3C) and within individual cells (Figure S3D). Notably, in GJ-connected astrocytes $[Ca^{2+}]$ did not depend on the distance from the patched cell (Figure 3A; Figure S3E), even though the OGB-1 concentration inside such cells must drop steeply with this distance (Henneberger et al., 2010). Furthermore, increasing OGB-1 in the patch pipette 2-fold had no effect on resting $[Ca^{2+}]$, in either patched or GJ-connected cells (Figure S3F), thus confirming little effect of the dye concentration on the astroglial basal $[Ca^{2+}]$.

Although our experiments dealt with quiescent tissue, astroglia in these conditions could still show spontaneous local

Ca^{2+} rises; in individual branches they appear at a frequency of less than one per minute in most cases, in situ or in vivo (Di Castro et al., 2011; Kanemaru et al., 2014). Because [Ca^{2+}] rises could potentially affect time-averaged basal [Ca^{2+}] maps, we separated branches that did and did not generate [Ca^{2+}] signals over the imaging period (termed active and inactive braches, respectively). Consistent with previous observations (Di Castro et al., 2011; Kanemaru et al., 2014), we could typically register one or fewer Ca^{2+} event over a 50–60 s period in the active branch (Figure S3G; the settings enabled both OGB-1 FLIM readout and intensity monitoring). We found that the transient Ca^{2+} rise, even when present, had a negligible (<6%) effect on the time-averaged [Ca^{2+}] measure over a typical 50 s exposure (Figures S3H and S3I); this small effect was further reduced if inactive branches were included into the time-averaged measurements. Thus, the typical spontaneous Ca^{2+} activity in our conditions had little effect on resting [Ca^{2+}].

Thus, dye diffusion loading through GJs, single-cell bolus loading, or in vivo multi-cell bolus loading enable robust [Ca^{2+}] FLIM measurements in largely unperturbed astroglia. Reassuringly, peripheral regions of the whole-cell-held astrocytes approach resting [Ca^{2+}] conditions characteristic for the periphery of unperturbed cells (Figure 3E).

Heterogeneous Patterns of Resting [Ca^{2+}] in Neuronal Dendrites

While activity-dependent rapid Ca^{2+} entry in neuronal axons or dendrites has been intensely studied, much less is known about their resting [Ca^{2+}]. The latter, however, is key to the local Ca^{2+} buffering capacity, by setting the occupancy level for endogenous Ca^{2+}-binding proteins (Grienberger and Konnerth, 2012; Ross, 2012; Rusakov, 2015). Remarkably, mapping resting [Ca^{2+}] at high resolution revealed significant heterogeneity of local [Ca^{2+}] within dendritic compartments of CA1 pyramidal cells (Figure 4A). Such heterogeneity could not be explained by random Ca^{2+} fluctuations because it could be consistently registered at least over several minutes (Figure 4B).

Sustained concentration gradients suggest that local [Ca^{2+}] results from a dynamic equilibrium between local Ca^{2+} sources, sinks, and buffers, rather than from diffusion equilibration along cell dendrites. Thus, resting [Ca^{2+}] might reflect the state or type of microscopically compartmentalized cell function. To follow up this line of thought, we asked if resting [Ca^{2+}] reflected the morphological identity of a local synapse. First, we examined [Ca^{2+}] distribution among dendritic spines of different sizes. The spine head volume estimator was a ratiometric measure (Figure S4A), which was insensitive to fluorescence signal intensity and appeared in good agreement with the recent STED microscopy estimate obtained in CA1 pyramidal cells (Tønnesen et al., 2014). Focusing on spines in higher-order branches (least likely to be affected by pipette dialysis), we found that intra-head [Ca^{2+}] varied substantially (mean ± SD; 83 ± 36 nM, n = 417) and was negatively correlated with the head volume (Figure 4C). This small, yet statistically significant, anti-correlation was not due to an unknown measurement bias, as the same approach applied in primary dendrites showed no such effect (Figure S4B). This result suggests that small-head (thin) spines, which reportedly contain predominantly NMDA receptors (Matsuzaki et al., 2001), tend to have higher basal [Ca^{2+}] compared to larger spines (that host both AMPA and NMDA receptors; Matsuzaki et al., 2001). Intriguingly, [Ca^{2+}] inside the spine head was consistently higher than [Ca^{2+}] in the adjacent dendritic shaft (by 8.0% ± 1.7%, n = 417, $p < 0.001$; dotted line in Figure 4D), and this difference did not depend on the head size (Figure 4D). However, Ca^{2+} gradients along the dendritic shaft appeared more substantial (20%–100% concentration difference range; Figures 4A and 4B). The autocorrelation analysis of [Ca^{2+}] along the dendrite suggested that the typical distance over which such gradients were sustained was 1.5–2 μm (Figure 4E).

Resting [Ca^{2+}] in Dendrites Shows Developmental and Activity-Dependent Plasticity

To test whether resting dendritic [Ca^{2+}] changes during development, we compared primary dendrites of CA1 pyramidal cells at P7, P15, and P21. Average [Ca^{2+}] was reduced approximately 2-fold between P7 and P15, with no further significant changes at P21 (Figure 4F). Consistent with this observation, previous studies pointed to developmental strengthening of intracellular Ca^{2+} buffering and pumping capacities in hippocampal neurons (Kip et al., 2006; Oh et al., 2013), which could also mechanistically explain shorter and sharper transients of free Ca^{2+} in older dendrites (Matthews et al., 2013; Pohle and Bischofberger, 2014) .

The observed developmental changes in dendritic [Ca^{2+}] prompted us to ask whether physiologically relevant cellular activities could affect steady-state basal dendritic [Ca^{2+}]. Surprisingly, we found that short bursts of back-propagating dendritic action potentials (4–6 spikes generated by 200 ms somatic depolarization steps, 1–3 steps in total, 10 s apart) led to a significant reduction of [Ca^{2+}] in dendritic shafts (on average from 59 ± 18 to 53 ± 13 nM, n = 109, $p < 0.001$) and in spine heads (79 ± 22 to 65 ± 15 nM, n = 145, $p < 0.001$) lasting for at least 5–10 min (Figures 4G and 4H). Interestingly, the activity-induced [Ca^{2+}] reduction in spine heads was consistently stronger than that in dendritic shafts (15% ± 2% compared to 6.0% ± 1.4%, respectively; $p < 0.001$). This observation suggests the existence of a rapidly occurring use-dependent plasticity mechanism involving postsynaptic Ca^{2+} homeostasis.

Astroglial Basal [Ca^{2+}] Is Age Dependent and Divides Cells into Two Sub-groups

Monitoring unperturbed GJ-connected astrocytes (Figure 3) has provided an opportunity to test whether their resting [Ca^{2+}] changes during development. First, we found that resting astroglial [Ca^{2+}] decreased dramatically within the week from P15 to P21 (Figure 5B; n = 23–44 and n = 53–79 astrocytes depending on recorded cell compartment; $p < 0.01$ throughout). Second, both ages showed the characteristic monotonic elevation of basal [Ca^{2+}] toward the cell periphery (Figures 5A and 5B).

While the average resting [Ca^{2+}] in GJ-connected astrocytes did not depend on the depth in tissue or the distance from the patch pipette (Figure S3E), it varied significantly among cells (Figure 5C). As noted above, this variation was not a consequence of intermittent Ca^{2+} activity or differences in intracellular OGB-1 concentrations. Nonetheless, the distribution of basal [Ca^{2+}] in the recorded astrocyte population showed at least two distinct sub-groups, one centered around 70–75 nM [Ca^{2+}]

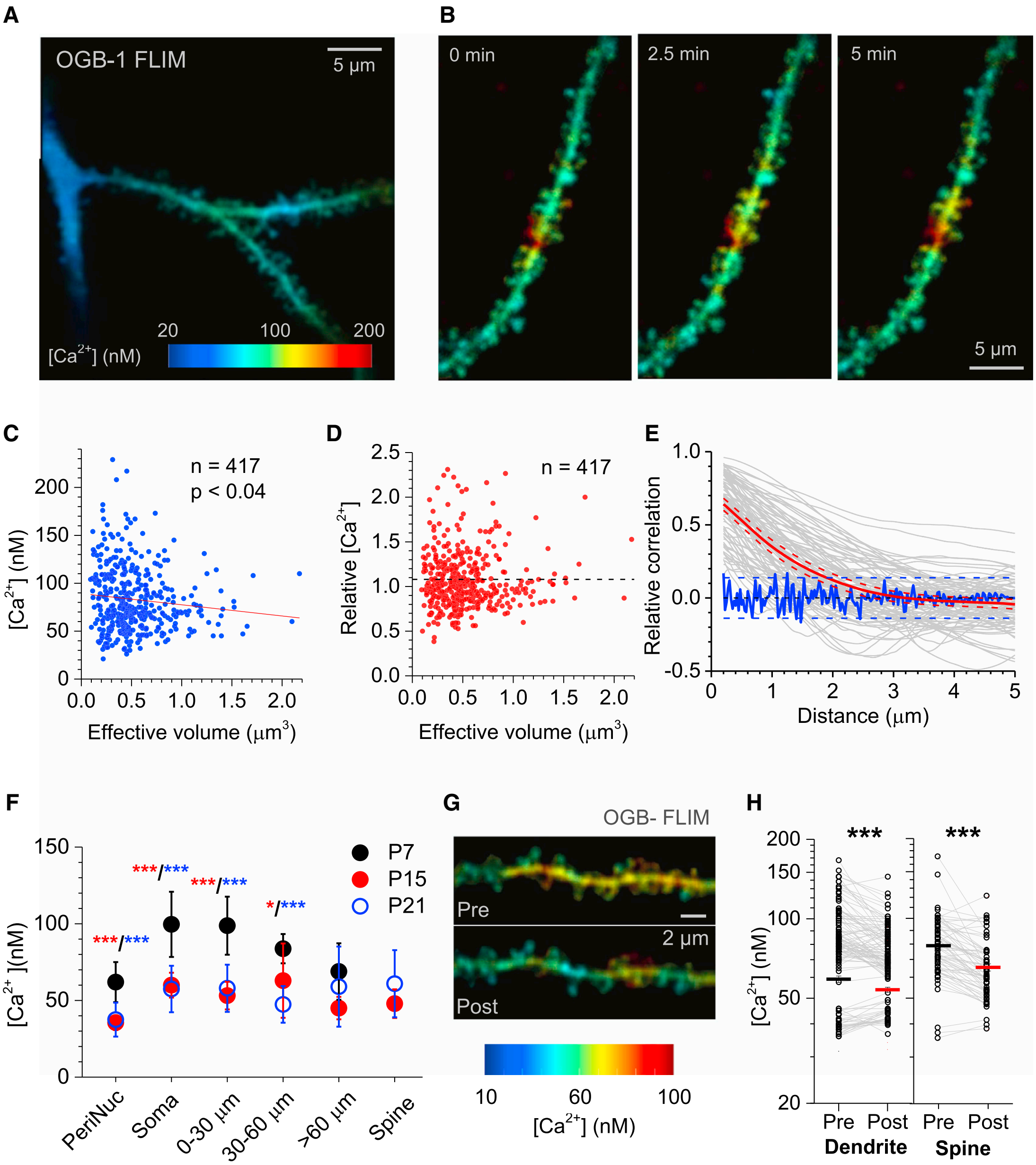

Figure 4. Uneven Distribution and Use-Dependent Plasticity of Resting [Ca^{2+}] in Dendritic Spines of CA1 Pyramidal Cells

(A) Example of a characteristic [Ca^{2+}] map (OGB-1 FLIM readout) for a dendritic branch in a hippocampal CA1 pyramidal cell; note significant [Ca^{2+}] gradients along the dendrites.

(B) An example illustrating dendritic [Ca^{2+}] gradients, which are sustained on the timescale of minutes (frame collection timing is shown); color-coded scale in (A) applies.

(C) Significant correlation (Pearson's r) between [Ca^{2+}] and the effective spine head volume; dotted line, significant negative regression.

(D) No correlation between the effective spine head volume and the spine – dendrite [Ca^{2+}] difference (relative values shown); dotted horizontal line, average spine – dendrite difference in [Ca^{2+}] (8.0% ± 1.7%, n = 417, $p < 0.001$).

(E) The autocorrelation-type function depicting relative [Ca^{2+}] values (ordinate) along the cell dendrite (abscissa); red line, mean ± 95% confidence limits; gray lines, functions on individual fragments (n = 90); blue line, characteristic noise function (obtained by pixel bootstrapping); blue dotted lines, noise 95% confidence intervals.

(F) Average [Ca^{2+}] (mean ± SEM) in different CA1 pyramidal cell compartments for three developmental stages, as indicated (P7, n = 6; P15, n = 13; P21, n = 44); *$p < 0.05$; ***$p < 0.001$ (shown in red for P7–P15 difference and in blue for P7–P21 difference; differences between other groups were insignificant).

(G) Characteristic example of a [Ca^{2+}] map (OGB-1 FLIM readout) in a spiny dendritic fragment, in resting conditions and ~5 min after a short burst of back-propagating spikes (see text for details); color-coded scale applies throughout.

(H) Statistical summary of experiments depicted in (G); dots, individual measurements in dendrites (n = 109) and spines (n = 145), as indicated; gray lines connect paired data points from the same experiments; horizontal bars, average values; ***$p < 0.001$.

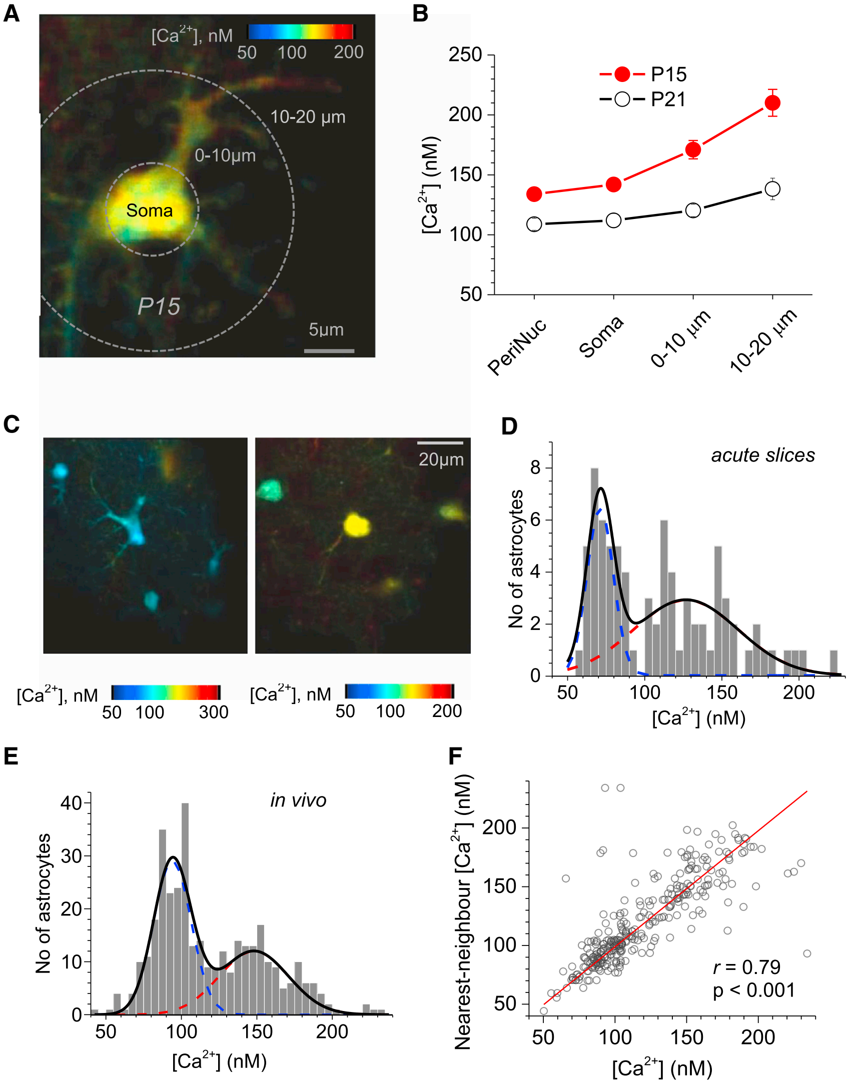

Figure 5. Basal [Ca^{2+}] in CA1 Astrocytes Reduces with Development and Shows Population Dichotomy

(A) Characteristic [Ca^{2+}] map in a GJ-connected (unperturbed) astrocyte at high resolution, at P15, with the regions of interest depicted.

(B) Distribution of resting [Ca^{2+}] in cellular compartments of GJ-connected astrocytes (mean ± SEM; regions of interests are as in A) at two developmental stages, as indicated (P15, n = 40; P21, n = 76).

(C) Characteristic examples of [Ca^{2+}] maps in two astrocyte groups imaged in one experiment and GJ connected (unperturbed) to one patched astrocyte in an acute slice. The two groups appear to differ in their average [Ca^{2+}], as shown.

(D) Frequency distribution of average basal [Ca^{2+}] in the recorded sample (n = 78 cells) of GJ-connected cells in slices; solid line, histogram best fit – the sum of two Gaussian distributions (dotted lines).

(E) Frequency distribution of average basal [Ca^{2+}] in the sample of SR101-labeled OGB-1-loaded cortical astrocytes in vivo (n = 357 cells, as shown in Figure 3D); other notations are as in (D).

(F) [Ca^{2+}] in the nearest neighbor plotted against basal [Ca^{2+}] in cortical astroglia in vivo. Strong positive correlation indicates strong neighborhood preference of astrocytes with similar basal [Ca^{2+}].

and the other, broader group centered at 120–130 [Ca^{2+}] nM (Figure 5D, the apparent boundary being at 80–90 nM). Remarkably, we identified a similar dichotomy in vivo (Figure 5E), albeit with the slightly elevated division boundary (100–120 nM, probably because of a greater contribution of spontaneous activity in the lower-[Ca^{2+}] group).

Relatively large numbers of imaged cells in vivo within contiguous regions of interest (n = 343) prompted us to ask whether these two [Ca^{2+}] sub-groups showed any spatial associations. Indeed, we found that basal [Ca^{2+}] values were highly correlated between the nearest astrocyte neighbors (Figure 5F), whereas the nearest-neighbor distances per se did not depend on basal astroglial [Ca^{2+}] (Figure S5A). This indicated that astrocytes with similar basal [Ca^{2+}] tended to group together, whereas spatial density of astroglia is not related to their resting [Ca^{2+}]. Conversely, when astrocytes were classified into the lower- and higher-[Ca^{2+}] groups, strong correlations indicated, once again, the prevalence of neighbors with similar [Ca^{2+}] levels (Figure S5B). It would be important to see whether this apparent dichotomy can be related to the two distinct functional states of local hippocampal neurons (Cossart et al., 2003; see Discussion).

DISCUSSION

In this study we have implemented and validated the method of monitoring low resting [Ca^{2+}] and [Ca^{2+}] dynamics in neurons and astroglia in situ at high resolution using two-photon excitation FLIM of the common Ca^{2+} indicator OGB-1. The FLIM-based [Ca^{2+}] readout (NTC) provides unprecedented [Ca^{2+}] sensitivity in the 10–200 nM range and is inherently insensitive to the dye concentration or depth of imaging in organized brain tissue. We have also found that this method of [Ca^{2+}] measurement is insensitive to all major concomitants of intracellular microenvironment.

Why is the knowledge about basal [Ca^{2+}] essential for understanding cell physiology that relies on Ca^{2+} signals? The level of free Ca^{2+} results from a dynamic equilibrium between local Ca^{2+} entry, removal, and binding-unbinding with local Ca^{2+} buffers. Thus, it is a key reflection of the Ca^{2+} homeostasis machinery present in a given cell compartment. Correspondingly, changes in resting [Ca^{2+}] could directly alter the availability (and thus buffering capacity) of Ca^{2+}-free endogenous Ca^{2+}-binding proteins; in physiologically relevant circumstances this relationship could be roughly proportional, even though changes in [Ca^{2+}] and changes in the available buffer concentration are likely to occur on the nanomolar and micromolar scales, respectively (Ruiz et al., 2003; Rusakov, 2015). Indeed, basal [Ca^{2+}] has been recognized to impact on the steady-state occupancy level

for Ca^{2+} sensors and Ca^{2+}-binding proteins (Grienberger and Konnerth, 2012; Ross, 2012), which in turn provides a mechanism to control the dynamics of rapid Ca^{2+} signals.

The present method has allowed us to test, in a relatively direct fashion, a long-standing question of whether whole-cell configuration significantly disturbs endogenous Ca^{2+} homeostasis in patched neurons and astrocytes in situ. We have found that, perhaps surprisingly, principal neurons are capable of maintaining what appears to be their endogenous low resting $[Ca^{2+}]$ throughout the patched cell. In contrast, astrocytes held in whole cell showed somewhat elevated $[Ca^{2+}]$, at least in their somatic proximity. However, because astroglia are heavily interconnected via GJs permeable to OGB-1, we could use our method to document $[Ca^{2+}]$ in relatively unperturbed GJ-connected astroglia, with submicron resolution. In addition, we have found that in both neurons and astroglia, a 2-fold change in the intracellular concentration of OGB-1 does not significantly affect endogenous $[Ca^{2+}]$. Importantly, our FLIM measurements of basal $[Ca^{2+}]$ in acute slices were entirely consistent with those made in vivo, also including bulk loading designs, which minimize disturbance of cell function. Because intracellular Ca^{2+} indicators are universally used, these observations lend support for the physiological relevance of such methods.

Perhaps unexpectedly, our approach has unveiled some previously unrecognized features of intracellular Ca^{2+} homeostasis that might have fundamental implications for cell function. First, it appears that resting $[Ca^{2+}]$ is heterogeneously distributed among cell compartments, in both neurons and in astroglia, showing sustained intracellular gradients. This is consistent with the notion that basal $[Ca^{2+}]$ is powerfully controlled by local cellular Ca^{2+} sinks and sources that enact a kinetic equilibrium for free Ca^{2+} in the vicinity. Physiologically relevant sinks and sources of cellular Ca^{2+} have long been a subject of intense studies. They are likely to include a variety of Ca^{2+} channels, such as TRPA1 (Shigetomi et al., 2012, 2013b), along with less-understood Ca^{2+} stores and pumps (Parpura and Verkhratsky, 2012; Ross, 2012), while several Ca^{2+} buffering proteins, such as calmodulin, neurogranin, presenilin, and septin, have also been implicated in $[Ca^{2+}]$ regulation on a small scale (Faas et al., 2011; Ho and Shen, 2011; Sharma et al., 2013; Zhabotinsky et al., 2006). With Ca^{2+} homeostasis playing a key role in various cellular devices, the local $[Ca^{2+}]$ maintenance machinery can, in theory, assign specific functions, or perhaps temporary functional states, to certain intracellular compartments. Indeed, we have found that the morphological identity of dendritic spines, which has previously been associated with the receptor identity of the resident CA3-CA1 synapse (Matsuzaki et al., 2001), is also correlated with the local $[Ca^{2+}]$. Intriguingly, glutamate-evoked postsynaptic Ca^{2+} entry in spines appears inversely proportional to the spine volume in vivo (Noguchi et al., 2011), also pointing to the relationship between resting $[Ca^{2+}]$, Ca^{2+} influx, and the functional spine identity.

We have also detected lasting decreases of resting dendritic $[Ca^{2+}]$ following brief cell excitation. This observation points to a potential mechanism for local Ca^{2+} homeostasis to contribute to the post-activity memory trace generated by the cell. While spike-evoked increases in presynaptic resting $[Ca^{2+}]$ have been associated with short-term release probability boost (Sylantyev et al., 2013; Wu and Saggau, 1994), use-dependent postsynaptic changes in resting $[Ca^{2+}]$ have hitherto been difficult to detect. Our results might help to advance this line of study. Similarly, the present finding that resting $[Ca^{2+}]$ is reduced during development across both neurons and astroglia can shed new light on what contributes to changes in Ca^{2+} homeostasis with aging (Toescu and Verkhratsky, 2007).

It has also transpired that resting $[Ca^{2+}]$ in principal hippocampal neurons is significantly lower, on average, than that in the surrounding astroglia. While this indicates that the mechanisms maintaining the equilibrated Ca^{2+} in neurons and astroglia could differ (Parpura and Verkhratsky, 2012; Ross, 2012), what constitutes such mechanisms remains to be elucidated. Finally, we have found that the average resting $[Ca^{2+}]$ is not homogenously distributed across the astroglial population. There are at least two distinct groups of astrocytes featuring relatively high and low resting $[Ca^{2+}]$ levels, in both acute hippocampal slices and cortical astroglia in vivo. It also appears that the lower- and the higher-$[Ca^{2+}]$ cell groups tend to occupy contiguous space domains. Interestingly, it has been reported that cortical neurons in situ appear to stochastically switch between an UP state (which is associated with lasting cell excitation and elevated intracellular $[Ca^{2+}]$) and a DOWN state (Cossart et al., 2003). This has been related to the synchronized excitation in a local neural network, and the activity of local astroglia has been implicated in the underlying mechanism (Poskanzer and Yuste, 2011). Whether this or similar phenomena can be associated with the subdivision of astrocytes with respect to their basal $[Ca^{2+}]$ remains an open and intriguing question.

Clearly, these observations represent only a first step in uncovering what could be an important relationship between basal Ca^{2+} levels and local cell function on the microscopic scale. Dedicated studies will be required in order to understand, first, what particular molecular mechanisms control the uneven intracellular distribution of resting $[Ca^{2+}]$, and second, whether and how this distribution, if anything, regulates cellular function at the level of individual intracellular compartments.

EXPERIMENTAL PROCEDURES

Time-Resolved Imaging In Situ: Two-Photon Excitation FLIM

Two-photon excitation by femtosecond infrared laser pulses was used to restrict excitation and emission collection to a thin focal excitation plane 50–110 μm deep into the slice (limited by surface debris and patching in DIC mode) and 50–250 μm deep in vivo (limited by surface damage from durectomy and fluorescent signal scattering). We ensured that no contaminating fluorescence signal was collected from damaged tissue near the slice surface, and no autofluorescence was detected at these depths (before applying OGB-1). Importantly, a short-pass 700 nm filter was placed in front of the detector to block out any escaped light from the laser source. The imaging system was based on a Biorad Radiance 2000 coupled with SPC-830 TCSPC Becker & Hickl imaging module under an Olympus LumplanFI 60× water immersion objective (NA 0.9) or an Olympus FV1000 coupled with Picoquant Picoharp 300 TCSPC system under Olympus XLPlan N 25× water immersion multi-photon objective (NA 1.05). Various digital zooms (2×–15×) were used to allow imaging of different cellular structures. The two-photon laser source was a Newport-Spectraphysics Ti:Sapphire MaiTai laser pulsing at 80 Mhz, with a pulse width of ~220 fs and a wavelength of 800 nm optimized for OGB-1 excitation. The laser power was kept below 8 mW under the objective at all times for slice preparations to minimize phototoxic damage. The laser power was also minimized for

in vivo imaging according to depth, where a maximum ~70 mW power under the objective was used for imaging depth at ~250 μm depth.

Fluorescence images were acquired at a laser line scanning rate of 500 lines per second and stored as a 256 × 256 × 256 × n (*t,x,y,T*) tensor representing *x-y* images with a distribution of nanosecond delay time (*t*) of photons at each pixel over the frame duration (*T*), which could also include z stack information. Average image acquisition times were 30–300 s (maximum laser exposure time of <100 s) depending on the total photon count, and the maximum photon count rate was on average $<10^5\ s^{-1}$, which is well below the effect of photon pile-up (maximal photon count of the system was near $10^8\ s^{-1}$). To monitor possible [Ca^{2+}] fluctuations, we minimized the sampled image area to approximately 6 × 3 μm (*x,y*) and a digital size of 100 × 50 pixels (*x,y*) so that a 10 Hz image acquisition rate could be achieved. Acquisition time of 10 s for each session was used, with >30 s resting period between each session, to minimize phototoxic damage. Because of relatively low photon counts per pixel, the photon data were accumulated over the entire image from (*t,x,y,T*) tensor into (*t,T*) matrix before the data analysis was used to estimate Ca^{2+} concentrations.

For various experimental purposes, the fluorescence intensity can always be calculated from FLIM data by integration of (non-normalized) photon counting data at any given pixel or ROI. Physical hardware (Becker & Hickl HPC-CON unit) can also be used to split the same signal stream from the photo-detector into digital (photon timing, FLIM) and analog (photon intensity) signal streams so that intensity and FLIM data can be acquired simultaneously in the same imaging system.

OGB-1 Calibration for [Ca^{2+}] Readout

The OGB-1 calcium calibration protocol was similar to the standard calibration method provided by the Invitrogen Ca^{2+} calibration buffer kit manual. However, to match the Ca^{2+} buffering dynamics to that of OGB-1 more closely, the standard 10 mM chelating agent EGTA was replaced with 10 mM BAPTA, and the solution constituents were replaced with our experimental intracellular solution (see below). pH was adjusted using KOH, and the KCl concentration in the intracellular solution was adjusted accordingly to keep ion constituents in the solution unchanged. The estimated [Ca^{2+}] was therefore slightly different from the standard Invitrogen's calibration set and was finely adjusted using Chris Patton's WEBMAXC program at Stanford University (http://web.stanford.edu/~cpatton/webmaxcS.htm). In the acidity control tests, pH was altered using KOH. [Mg^{2+}] and [Zn^{2+}] were changed using $MgCl_2$ and $ZnCl_2$, respectively, whereas other constituents were adjusted to maintain the original composition of the intracellular solution. Actin protein calibration was adjusted using Actin from Bovine Muscle (Sigma A3653), temperature was varied (from 19°C to 37°C) using a Scientific Systems Design PTC03 in-line heater, and solution viscosity was varied by adding 70 kDa Dextran to ACSF or internal solution.

Time-Resolved Imaging In Situ: FLIM Data Analyses and Mapping

The fluorescent decay time course was first normalized to its peak value and then integrated (area under curve) over 9 ns post-peak (Figure S1A). The resulting value was termed normalized total count (NTC). We used this parameter throughout as an estimator for [Ca^{2+}] using an appropriate calibration function obtained through direct OGB-1 calibration for intracellular solutions of clamped [Ca^{2+}], as further explained in the text.

For illustration purposes, [Ca^{2+}]-mapping pixel values were spatially averaged using 5–10 nearest-neighbor pixels to ensure that the FLIM decay traces had at least 10 counts near the tail. Any pixel that did not satisfy such criteria was not calculated as a valid data point and discarded (depicted in images as blank pixels). All the statistics and metrics for individual cellular compartments were, however, obtained from the raw data matrices, not from the smoothed mapped images. To maximize the measurement accuracy and reliability for individual selected cell compartments, we used photon count information within this region only and averaged the data to give an overall [Ca^{2+}] estimate for that compartment.

Time-Resolved Imaging In Situ: Fast FLIM

To enable monitoring of rapid Ca^{2+} transients in neuronal processes, we sought to maximize the FLIM readout acquisition rate. We therefore further modified our experimental procedures to gain maximum temporal resolution by sacrificing most of the spatial information. Because we based our FLIM decay analysis on photon count integration rather than on multi-exponential fitting (see the text and below), the requirement to the time resolution of the OGB-1 fluorescence decay (on the nanoscopic scale) was less stringent. This implied a reduction in the total number of photons required for the decay trace; hence, less average exposure time was required. To image a selected dendritic fragment, line scan at the rate of 750 Hz (lines per second) was run across a small segment of the dendritic stem (CA1 pyramidal cell apical dendrites) and the associated spine for 2 s. The resulting FLIM photon count data were averaged over progressively longer periods of scanning: we found that in characteristic imaging experiments in acute brain slices the time window of 6–10 ms per [Ca^{2+}] data point was sufficient to meet the analysis criteria for reliable [Ca^{2+}] readout (see below). In our conditions we thus were able to increase the FLIM acquisition frequency to 100 Hz.

Preparation of Acute Slices

Acute hippocampal transverse slices (350 μm thick) were prepared from P7–P8, P15–P16, and P21–P24 Sprague-Dawley rats, in full compliance with the national guidelines, the European Communities Council Directive of 24 November 1986, and the European Directive 2010/63/EU on the Protection of Animals used for Scientific Purposes. Slices were prepared in an ice-cold slicing solution containing (in mM) NaCl 60, sucrose 105, $NaHCO_3$ 26, KCl 2.5, NaH_2PO_4 1.25, $MgCl_2$ 7, $CaCl_2$ 0.5, glucose 11, ascorbic acid 1.3, and sodium pyruvate 3 (osmolarity 300–310 mOsM), stored in the slicing solution at 34°C for 15 min, and transferred for storage in an extracellular solution containing (in mM) NaCl 125, $NaHCO_3$ 26, KCl 2.5, NaH_2PO_4 1.25, $MgSO_4$ 1.3, $CaCl_2$ 2, and glucose 16 (osmolarity 300–305 mOsm). All solutions were continuously bubbled with 95% O_2/5% CO_2. Slices were allowed to rest for at least 60 min before recordings started.

Electrophysiology and Dye Loading

Whole-cell patch-clamp recordings of CA1 pyramidal cells and *stratum radiatum* astrocytes were performed in a submersion-type recording chamber. Slices were superfused with an extracellular solution containing (in mM) NaCl 125, $NaHCO_3$ 26, KCl 2.5, NaH_2PO_4 1.25, $MgSO_4$ 1.3, $CaCl_2$ 2, and glucose 16 (osmolarity 300–305 mOsm), continuously bubbled with 95% O_2/5% CO_2. Whole-cell recordings were obtained with patch pipettes (3–5 MΩ) with an intracellular solution containing (in mM) KCH_3O_3S 135, HEPES 10, Tris-phosphocreatine 10, $MgCl_2$ 4, Na_2ATP 4, and Na_3GTP 0.4 (pH adjusted to 7.2 with KOH, osmolarity 290–295 mOsM). The cell impermeable Ca^{2+} indicator OGB-1 (200 μM unless indicated otherwise; Invitrogen O6806) was added to the internal solution. CA1 pyramidal cells were held at −70 mV. Protoplasmic astrocytes located in the *stratum radiatum* were identified by their small soma size, low resting potential (<−80 mV), and low input resistance (<10 MΩ). Astrocytes were held in voltage clamp at their resting potential or in current clamp.

In single-cell bolus-loading experiments, cells were held in whole-cell mode for only 1–3 min, after which the pipette was carefully withdrawn until an outside-out configuration was obtained to allow the somatic membrane to seal safely. [Ca^{2+}] FLIM measurements were carried out across the dendritic tree several minutes after the re-seal to minimize diffusion escape of the dye.

In Vivo Preparation and Cranial Window Implantation

All in vivo animal experiments were conducted using male Sprague-Dawley rats (p27–P33) in accordance with the European Commission Directive (86/609/EEC) and the United Kingdom Home Office (Scientific Procedures) Act (1986).

Young male rats (100–120 g) were anesthetized with urethane (initial dose 1.3 g/kg, i.p.; then 10–25 mg/kg/hr, i.v.) following isoflurane (5% in air) induction. Adequate anesthesia was ensured by maintaining stable levels of the arterial blood pressure and heart rate showing lack of responses to a paw pinch. The femoral artery and vein were cannulated for measurement of the arterial blood pressure and administration of anesthetic, respectively. The trachea was cannulated, and the animal was ventilated with O_2-enriched room air using a positive pressure ventilator with a tidal volume of ~1 ml/100 g and a ventilator frequency similar to the resting respiratory rate (~60 strokes/min). The animal was then placed in a stereotaxic frame. The skin overlying the skull was removed, and a small craniotomy (~2 mm^2) was made in the parietal bone above the somatosensory cortex. Cortical astrocytes were labeled with sulforhodamine 101 (SR101) and OGB-1. The solution containing

OGB-1 (1 mM) and SR101 (to aid identification of astroglia, 25 μM) in artificial cerebrospinal fluid (aCSF; 124 mM NaCl, 3 mM KCl, 2 mM $CaCl_2$, 26 mM $NaHCO_3$, 1.25 mM NaH_2PO_4, 1 mM $MgSO_4$, 10 mM D-glucose saturated with 95% O_2/ 5% CO_2 [pH 7.4]) was delivered (volume 1.5 μl) via a glass micropipette to the targeted area of the right primary somatosensory cortex, immediately caudal of the coronal suture (~0–3 mm posterior to bregma and 2–5 mm lateral from the midline). The exposed surface of the cortex was then covered with 1% agarose and protected with a glass coverslip secured to the skull using acrylic dental cement.

Throughout surgical preparation and imaging, body temperature was maintained at 37.0°C. PO_2, PCO_2, and pH of the arterial blood were periodically measured using a RAPIDLab 348EX blood gas analyzer (Siemens Healthcare), and the ventilation parameters were adjusted to keep these variables within the physiological ranges. Two-photon excitation for FLIM acquisition was carried out as described below using a Newport-Spectraphysics Ti:Sapphire Mai-Tai laser, Olympus FV1000 with XLPlan N 25× water immersion multi-photon objective (NA 1.05), and Picoquant Picoharp 300 TCSPC. Acquisitions were carried out at a depth between 50 and 300 μm from the cortical surface.

Estimation of Spine Head Volume Based on Fluorophore Intensity

The principal assumption for spine head volume (V_s) estimate (based on the intensity readout for the cell-impermeable, water-soluble indicator) is that the fluorophore concentration and excitation laser power are uniform within the distal dendritic branches and nearby spines monitored in the same focal plane. The cytosolic volume of the compartment is therefore proportional to the fluorescent intensity observed, i.e., $V_s / V_d = I_s / I_d$, where index d stands for dendritic shaft values. The ROI (in the x-y plane) for V_d in the dendritic shaft was, however, selected to be as small as the smallest spine in the same image. This corresponded to the dendritic shaft volume, which was smaller than the shaft diameter, thus providing 100% sampled emission represented by the fluorophore, also mitigating the effect of boundaries and volume fractions (Figure S4A). In other words, V_d represented the average fluorescence intensity over the depth of the system's point spread function (PSF), whereas V_s represented a fraction of this intensity over the same depth, thus directly reflecting the structure volume. Because the system PSF was unchanged throughout the experiments, V_s / V_d provided a fully consistent and unbiased relative volume measure. To arrive at the absolute volume scale, we used the characteristic PSF depth at 800 nm (Scott and Rusakov, 2006); this value, however, was not important for any comparative analyses.

Statistical Methods

The statistical data in graphs were normally presented as mean ± SEM or mean ± SD, where indicated. We routinely used two-sided t tests to compare sample averages with respect to the null hypothesis, and for non-Guassian data scatters the non-parametric Mann-Whitney test was used. These tests, as well as Spearman's correlation r, linear regression, and autocorrelation analyses, were used as implemented in Origin (Origin Lab Corp).

SUPPLEMENTAL INFORMATION

Supplemental Information includes five figures and one movie and can be found with this article online at http://dx.doi.org/10.1016/j.neuron.2015.09.043.

AUTHOR CONTRIBUTIONS

K.Z. designed and implemented the FLIM-based method, including imaging and analysis protocols; L.B. carried out patch-clamp and imaging experiments in neurons and glia; T.P.J. carried out some patch-clamp and imaging experiments in neurons; A.V.G, J.R., K.Z., and C.K. designed and carried out in vivo experiments; and D.A.R. narrated the study, which was subsequently contributed to by all the authors.

ACKNOWLEDGMENTS

This work was supported by the Wellcome Trust Principal (D.A.R.) and Senior (A.V.G.) Fellowships, Medical Research Council (UK), Biology and Biotechnology Research Council (UK), European Research Council Advanced Grant, FP7 ITN EXTRABRAIN grant, Russian Science Foundation grant 15-14-30000 (D.A.R.), and EMBO Fellowship (L.B.). The authors thank Klaus Suhling, Dimitri Kullmann, and George Augustine for insightful comments on preliminary results.

Received: January 1, 2015
Revised: August 1, 2015
Accepted: September 17, 2015
Published: October 21, 2015

REFERENCES

Baker, A.J., Brandes, R., Schreur, J.H.M., Camacho, S.A., and Weiner, M.W. (1994). Protein and acidosis alter calcium-binding and fluorescence spectra of the calcium indicator indo-1. Biophys. J. *67*, 1646–1654.

Battisti, A., Digman, M.A., Gratton, E., Storti, B., Beltram, F., and Bizzarri, R. (2012). Intracellular pH measurements made simple by fluorescent protein probes and the phasor approach to fluorescence lifetime imaging. Chem. Commun. (Camb.) *48*, 5127–5129.

Baylor, S.M., and Hollingworth, S. (2011). Calcium indicators and calcium signalling in skeletal muscle fibres during excitation-contraction coupling. Prog. Biophys. Mol. Biol. *105*, 162–179.

Cossart, R., Aronov, D., and Yuste, R. (2003). Attractor dynamics of network UP states in the neocortex. Nature *423*, 283–288.

Di Castro, M.A., Chuquet, J., Liaudet, N., Bhaukaurally, K., Santello, M., Bouvier, D., Tiret, P., and Volterra, A. (2011). Local Ca2+ detection and modulation of synaptic release by astrocytes. Nat. Neurosci. *14*, 1276–1284.

Ermolyuk, Y.S., Alder, F.G., Henneberger, C., Rusakov, D.A., Kullmann, D.M., and Volynski, K.E. (2012). Independent regulation of basal neurotransmitter release efficacy by variable Ca^{2}+ influx and bouton size at small central synapses. PLoS Biol. *10*, e1001396.

Faas, G.C., Raghavachari, S., Lisman, J.E., and Mody, I. (2011). Calmodulin as a direct detector of Ca2+ signals. Nat. Neurosci. *14*, 301–304.

Garaschuk, O., Milos, R.I., and Konnerth, A. (2006). Targeted bulk-loading of fluorescent indicators for two-photon brain imaging in vivo. Nat. Protoc. *1*, 380–386.

Gee, J.M., Smith, N.A., Fernandez, F.R., Economo, M.N., Brunert, D., Rothermel, M., Morris, S.C., Talbot, A., Palumbos, S., Ichida, J.M., et al. (2014). Imaging activity in neurons and glia with a Polr2a-based and cre-dependent GCaMP5G-IRES-tdTomato reporter mouse. Neuron *83*, 1058–1072.

Giaume, C., Koulakoff, A., Roux, L., Holcman, D., and Rouach, N. (2010). Astroglial networks: a step further in neuroglial and gliovascular interactions. Nat. Rev. Neurosci. *11*, 87–99.

Grienberger, C., and Konnerth, A. (2012). Imaging calcium in neurons. Neuron *73*, 862–885.

Grynkiewicz, G., Poenie, M., and Tsien, R.Y. (1985). A new generation of Ca2+ indicators with greatly improved fluorescence properties. J. Biol. Chem. *260*, 3440–3450.

Halassa, M.M., and Haydon, P.G. (2010). Integrated brain circuits: astrocytic networks modulate neuronal activity and behavior. Annu. Rev. Physiol. *72*, 335–355.

Henneberger, C., Papouin, T., Oliet, S.H., and Rusakov, D.A. (2010). Long-term potentiation depends on release of D-serine from astrocytes. Nature *463*, 232–236.

Ho, A., and Shen, J. (2011). Presenilins in synaptic function and disease. Trends Mol. Med. *17*, 617–624.

Kanemaru, K., Sekiya, H., Xu, M., Satoh, K., Kitajima, N., Yoshida, K., Okubo, Y., Sasaki, T., Moritoh, S., Hasuwa, H., et al. (2014). In vivo visualization of subtle, transient, and local activity of astrocytes using an ultrasensitive Ca(2+) indicator. Cell Rep. *8*, 311–318.

Katz, B., and Miledi, R. (1968). The role of calcium in neuromuscular facilitation. J. Physiol. *195*, 481–492.

Kip, S.N., Gray, N.W., Burette, A., Canbay, A., Weinberg, R.J., and Strehler, E.E. (2006). Changes in the expression of plasma membrane calcium extrusion systems during the maturation of hippocampal neurons. Hippocampus *16*, 20–34.

Koester, H.J., Baur, D., Uhl, R., and Hell, S.W. (1999). Ca2+ fluorescence imaging with pico- and femtosecond two-photon excitation: signal and photodamage. Biophys. J. *77*, 2226–2236.

Koldenkova, V.P., and Nagai, T. (2013). Genetically encoded Ca2+ indicators: Properties and evaluation. Biochimica Et Biophysica Acta-Molecular Cell Research *1833*, 1787–1797.

Konishi, M., Olson, A., Hollingworth, S., and Baylor, S.M. (1988). Myoplasmic binding of fura-2 investigated by steady-state fluorescence and absorbance measurements. Biophys. J. *54*, 1089–1104.

Kuchibhotla, K.V., Lattarulo, C.R., Hyman, B.T., and Bacskai, B.J. (2009). Synchronous hyperactivity and intercellular calcium waves in astrocytes in Alzheimer mice. Science *323*, 1211–1215.

Lisman, J. (1989). A mechanism for the Hebb and the anti-Hebb processes underlying learning and memory. Proc. Natl. Acad. Sci. USA *86*, 9574–9578.

Maravall, M., Mainen, Z.F., Sabatini, B.L., and Svoboda, K. (2000). Estimating intracellular calcium concentrations and buffering without wavelength ratioing. Biophys. J. *78*, 2655–2667.

Matsuzaki, M., Ellis-Davies, G.C., Nemoto, T., Miyashita, Y., Iino, M., and Kasai, H. (2001). Dendritic spine geometry is critical for AMPA receptor expression in hippocampal CA1 pyramidal neurons. Nat. Neurosci. *4*, 1086–1092.

Matthews, E.A., Schoch, S., and Dietrich, D. (2013). Tuning local calcium availability: cell-type-specific immobile calcium buffer capacity in hippocampal neurons. J. Neurosci. *33*, 14431–14445.

Nishiyama, M., Hong, K., Mikoshiba, K., Poo, M.M., and Kato, K. (2000). Calcium stores regulate the polarity and input specificity of synaptic modification. Nature *408*, 584–588.

Noguchi, J., Nagaoka, A., Watanabe, S., Ellis-Davies, G.C.R., Kitamura, K., Kano, M., Matsuzaki, M., and Kasai, H. (2011). In vivo two-photon uncaging of glutamate revealing the structure-function relationships of dendritic spines in the neocortex of adult mice. J. Physiol. *589*, 2447–2457.

Oertner, T.G., Sabatini, B.L., Nimchinsky, E.A., and Svoboda, K. (2002). Facilitation at single synapses probed with optical quantal analysis. Nat. Neurosci. *5*, 657–664.

Oh, M.M., Oliveira, F.A., Waters, J., and Disterhoft, J.F. (2013). Altered calcium metabolism in aging CA1 hippocampal pyramidal neurons. J. Neurosci. *33*, 7905–7911.

Oheim, M., Beaurepaire, E., Chaigneau, E., Mertz, J., and Charpak, S. (2001). Two-photon microscopy in brain tissue: parameters influencing the imaging depth. J. Neurosci. Methods *111*, 29–37.

Otsu, Y., Couchman, K., Lyons, D.G., Collot, M., Agarwal, A., Mallet, J.-M., Pfrieger, F.W., Bergles, D.E., and Charpak, S. (2015). Calcium dynamics in astrocyte processes during neurovascular coupling. Nat. Neurosci. *18*, 210–218.

Parpura, V., and Verkhratsky, A. (2012). Homeostatic function of astrocytes: Ca(2+) and Na(+) signalling. Transl. Neurosci. *3*, 334–344.

Perea, G., Navarrete, M., and Araque, A. (2009). Tripartite synapses: astrocytes process and control synaptic information. Trends Neurosci. *32*, 421–431.

Pohle, J., and Bischofberger, J. (2014). Supralinear dendritic Ca(2+) signalling in young developing CA1 pyramidal cells. J. Physiol. *592*, 4931–4949.

Poskanzer, K.E., and Yuste, R. (2011). Astrocytic regulation of cortical UP states. Proc. Natl. Acad. Sci. USA *108*, 18453–18458.

Ross, W.N. (2012). Understanding calcium waves and sparks in central neurons. Nat. Rev. Neurosci. *13*, 157–168.

Ruiz, A., Fabian-Fine, R., Scott, R., Walker, M.C., Rusakov, D.A., and Kullmann, D.M. (2003). GABAA receptors at hippocampal mossy fibers. Neuron *39*, 961–973.

Rusakov, D.A. (2015). Disentangling calcium-driven astrocyte physiology. Nat. Rev. Neurosci. *16*, 226–233.

Scott, R., and Rusakov, D.A. (2006). Main determinants of presynaptic Ca^{2+} dynamics at individual mossy fiber-CA3 pyramidal cell synapses. J. Neurosci. *26*, 7071–7081.

Sharma, S., Quintana, A., Findlay, G.M., Mettlen, M., Baust, B., Jain, M., Nilsson, R., Rao, A., and Hogan, P.G. (2013). An siRNA screen for NFAT activation identifies septins as coordinators of store-operated Ca2+ entry. Nature *499*, 238–242.

Shigetomi, E., Tong, X., Kwan, K.Y., Corey, D.P., and Khakh, B.S. (2012). TRPA1 channels regulate astrocyte resting calcium and inhibitory synapse efficacy through GAT-3. Nat. Neurosci. *15*, 70–80.

Shigetomi, E., Bushong, E.A., Haustein, M.D., Tong, X., Jackson-Weaver, O., Kracun, S., Xu, J., Sofroniew, M.V., Ellisman, M.H., and Khakh, B.S. (2013a). Imaging calcium microdomains within entire astrocyte territories and endfeet with GCaMPs expressed using adeno-associated viruses. J. Gen. Physiol. *141*, 633–647.

Shigetomi, E., Jackson-Weaver, O., Huckstepp, R.T., O'Dell, T.J., and Khakh, B.S. (2013b). TRPA1 channels are regulators of astrocyte basal calcium levels and long-term potentiation via constitutive D-serine release. J. Neurosci. *33*, 10143–10153.

Sylantyev, S., Jensen, T.P., Ross, R.A., and Rusakov, D.A. (2013). Cannabinoid- and lysophosphatidylinositol-sensitive receptor GPR55 boosts neurotransmitter release at central synapses. Proc. Natl. Acad. Sci. USA *110*, 5193–5198.

Toescu, E.C., and Verkhratsky, A. (2007). The importance of being subtle: small changes in calcium homeostasis control cognitive decline in normal aging. Aging Cell *6*, 267–273.

Tønnesen, J., Katona, G., Rózsa, B., and Nägerl, U.V. (2014). Spine neck plasticity regulates compartmentalization of synapses. Nat. Neurosci. *17*, 678–685.

Tsien, R.Y. (1989). Fluorescent probes of cell signaling. Annu. Rev. Neurosci. *12*, 227–253.

Tsuji, T., Yoshida, S., Yoshida, A., and Uchiyama, S. (2013). Cationic fluorescent polymeric thermometers with the ability to enter yeast and mammalian cells for practical intracellular temperature measurements. Anal. Chem. *85*, 9815–9823.

Ueda, Y., Kwok, S., and Hayashi, Y. (2013). Application of FRET probes in the analysis of neuronal plasticity. Front. Neural Circuits *7*, 163.

Volterra, A., and Meldolesi, J. (2005). Astrocytes, from brain glue to communication elements: the revolution continues. Nat. Rev. Neurosci. *6*, 626–640.

Wilms, C.D., and Eilers, J. (2007). Photo-physical properties of Ca2+-indicator dyes suitable for two-photon fluorescence-lifetime recordings. J. Microsc. *225*, 209–213.

Wilms, C.D., Schmidt, H., and Eilers, J. (2006). Quantitative two-photon Ca2+ imaging via fluorescence lifetime analysis. Cell Calcium *40*, 73–79.

Wu, L.G., and Saggau, P. (1994). Presynaptic calcium is increased during normal synaptic transmission and paired-pulse facilitation, but not in long-term potentiation in area CA1 of hippocampus. J. Neurosci. *14*, 645–654.

Wu, Y., Stefl, M., Olzyńska, A., Hof, M., Yahioglu, G., Yip, P., Casey, D.R., Ces, O., Humpolíčková, J., and Kuimova, M.K. (2013). Molecular rheometry: direct determination of viscosity in Lo and Ld lipid phases via fluorescence lifetime imaging. Phys. Chem. Chem. Phys. *15*, 14986–14993.

Zhabotinsky, A.M., Camp, R.N., Epstein, I.R., and Lisman, J.E. (2006). Role of the neurogranin concentrated in spines in the induction of long-term potentiation. J. Neurosci. *26*, 7337–7347.

Zheng, K., Levitt, J.A., Suhling, K., and Rusakov, D.A. (2014). Monitoring Nanoscale Mobility of Small Molecules in Organized Brain Tissue with Time-Resolved Fluorescence Anisotropy Imaging. In Nanoscale Imaging of Synapses (Springer), pp. 125–143.

Neuron, Volume *88*

Supplemental Information

Time-Resolved Imaging Reveals Heterogeneous Landscapes of Nanomolar Ca^{2+} in Neurons and Astroglia

Kaiyu Zheng, Lucie Bard, James P. Reynolds, Claire King, Thomas P. Jensen, Alexander V. Gourine, and Dmitri A. Rusakov

SUPPLEMENTARY FIGURES

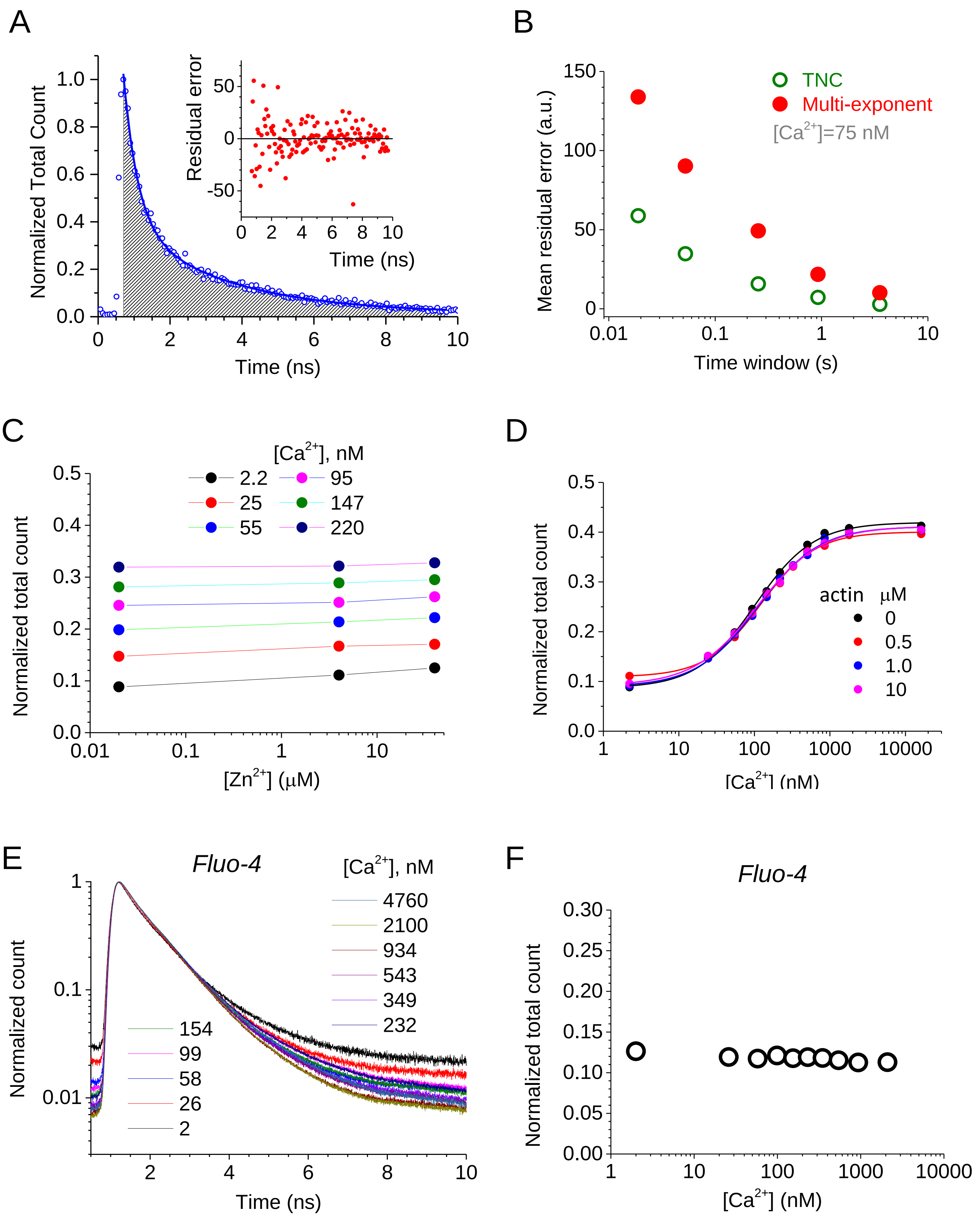

Figure S1 (Related to Fig. 1). Optimisation and concomitant sensitivity tests for the [Ca^{2+}]-sensitive OGB-1 fluorescence decay time course.

(A) Quantification of the OGB-1 fluorescence decay using Normalised Total Count (NTC) method *versus* double-exponent (DE) fitting method. Graph, example fluorescence decay trace (75 nM [Ca^{2+}] calibration solution); blue circles, raw photon count data; shaded area, integration area for NTC; blue curve, DE fitting (interval show by shade). Inset, plot of residuals (red dots) illustrating the fitting error for the DE method.

(B) Average error for [Ca^{2+}] estimation with FLIM (75 nM [Ca^{2+}] calibration solution) using the NTC (hollow green circles) or the DE (solid red circles) method with respect to different acquisition times (integration periods). As expected, increasing the acquisition time reduces the noise in the FLIM decay traces. Because of the higher sensitivity of the (non-linear) DE approximation to the experimental noise compared to the (linear) NTC approach, its overall error consistently exceeds that of the NTC method; a.u., arbitrary units.

(C) The sensitivity of OGB-1 fluorescence lifetime to [Ca^{2+}] at various free [Ca^{2+}] levels (using calibrated Ca^{2+} solutions, as indicated) is negligibly affected by [Zn^{2+}] in the 0.01-4 μM range.

(D) The sensitivity of OGB-1 fluorescence lifetime to [Ca^{2+}] is not affected by the presence of (a ubiquitous neuronal protein) actin over the range of 0-10 μM.

(E) The Fluo-4 fluorescence decay (photon count data) in calibrated solutions of concentration-clamped [Ca^{2+}], as indicated.

(F) Normalized total photon count ([Ca^{2+}] readout) for experiments in E shows little sensitivity of Fluo-4 to [Ca^{2+}].

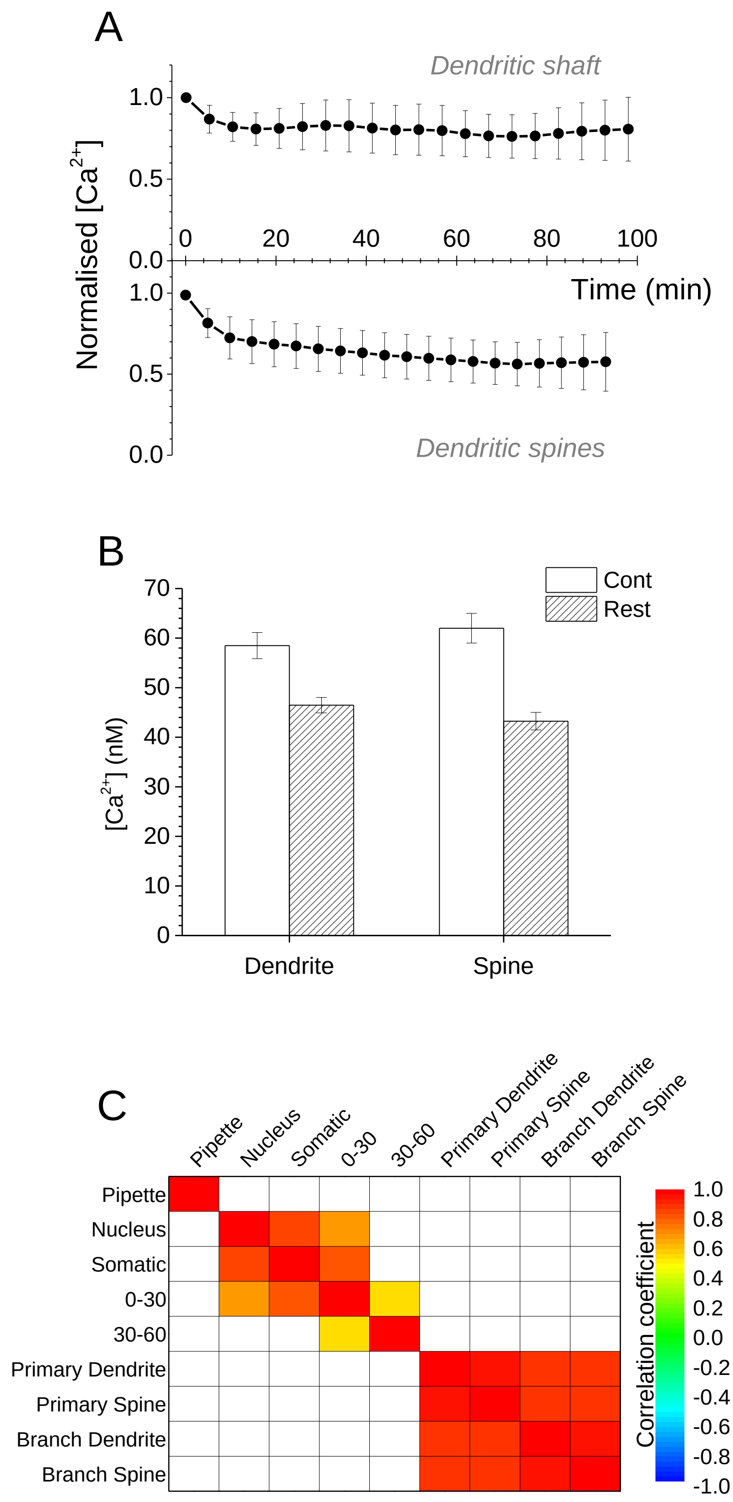

Figure S2 (Related to Fig. 2). Testing [Ca^{2+}] in CA1 pyramidal cells readout at varied imaging conditions.

(A) Time course of average resting [Ca^{2+}] (Normalized photon count for OGB-1 FLIM readout) after whole-cell break-in, in dendritic shafts and spines of CA1

pyramidal cells, as indicated. Dots (mean ± SEM), values averaged over multiple regions of interest throughout the dendritic tree in n = 3 cells.

(B) Average [Ca^{2+}] values (mean ± SEM) for two modes of image acquisition, continuous (Cont, n = 77) and with 3 second intervals between individual frames (Rest, n = 109 frames). Further increases in the inter-frame interval had no effect; one-cell example.

(C) Correlation coefficients for [Ca^{2+}] values across various cell compartments among cells in an experimental sample of n = 35, as indicated. Only statistically significant ($p < 0.05$) Pearson's correlation values are shown, empty table cells correspond to insignificant correlation.

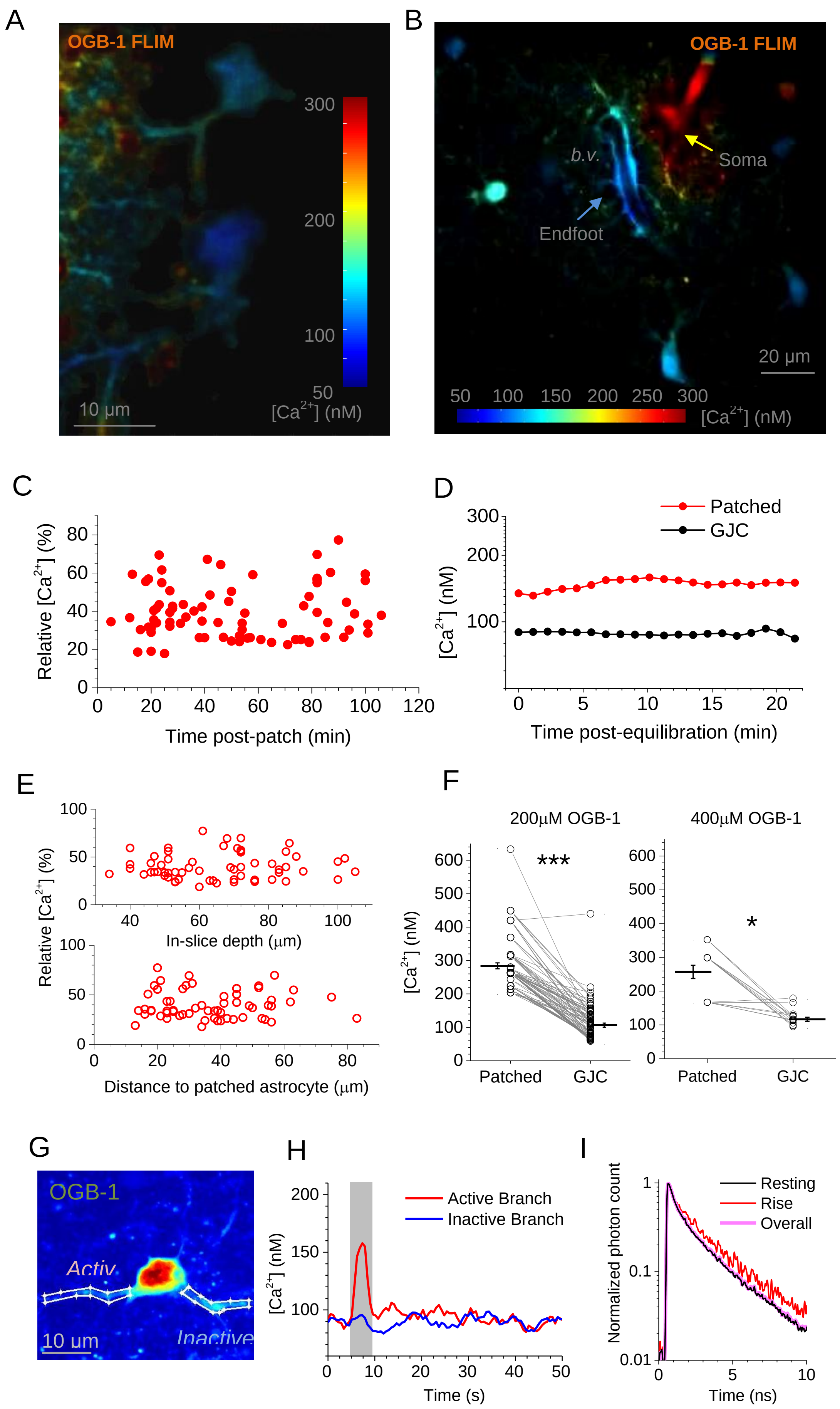

A
OGB-1 FLIM
300
200
100
50
10 µm
[Ca2+] (nM)
B
OGB-1 FLIM
b.v.
Soma
Endfoot
20 µm
50 100 150 200 250 300
[Ca2+] (nM)
C
Relative [Ca2+] (%)
Time post-patch (min)
D
Patched
GJC
[Ca2+] (nM)
Time post-equilibration (min)
E
Relative [Ca2+] (%)
In-slice depth (µm)
Distance to patched astrocyte (µm)
F
200µM OGB-1
400µM OGB-1
***
*
[Ca2+] (nM)
Patched
GJC
G
OGB-1
Activ
10 um
Inactive
H
Active Branch
Inactive Branch
[Ca2+] (nM)
Time (s)
I
Resting
Rise
Overall
Normalized photon count
Time (ns)

Figure S3 (related to Fig. 3). Monitoring basal [Ca^{2+}] in patched (whole-cell) and gap junction (GJ) -connected astroglia with OGB-1 FLIM readout.

(A) Examples of two GJ-connected astrocytes with false color-mapping for [Ca^{2+}] using OGB-1 NTC readout. A significant [Ca^{2+}] gradient between the somata and cell arbor peripheries can be seen, with resting [Ca^{2+}] values rising towards the periphery (in contrast to what is seen in a patched cell); see Fig. 3E for summary statistics.

(B) In a patched astrocyte (yellow arrow, patch pipette tip), the endfoot surrounding a blood vessel (*b.v.*, blue arrow) shows a 2-3 times lower [Ca^{2+}] than that near the soma; an example.

(C) Average [Ca^{2+}] in GJ-connected astrocytes does not depend on the time lapsed after cell patching, i.e. duration of dye diffusion through GJs (average for n = 78 cells).

(D) An example documenting the time course of average [Ca^{2+}] in one patched and one GJ-connected astrocyte (somatic area), as indicated, over the experiment duration.

(E) Average [Ca^{2+}] in GJ-connected astrocytes does not depend on the recording depth in slice (upper graph; dots, individual cell data; n = 62) or on the distance from the patched astrocyte (lower graph; n = 61); [Ca^{2+}] values shown relative to [Ca^{2+}] in the patched astrocyte soma.

(F) A two-fold increase in the loaded OGB-1 concentration has no effect on resting [Ca^{2+}], in either patched or GJ-connected astroglia, nor on the average [Ca^{2+}] difference between the two groups (somatic areas analyzed). Dots, individual cell data (n = 79 and n = 16 cells for 200 μM and 400 μM OGB-1, respectively); horizontal bars, mean ± SEM; *$p < 0.05$, ***$p < 0.001$.

(G) Example image (OGB-1 intensity, false color scale, planar projection) of an astrocyte with two delineated regions of interest in two (primary) cell branches, one of which (*Active*) did while the other (*Inactive*) did not generate a spontaneous [Ca^{2+}] rise during the recording period of 50 s.

(H) OGB-1 NTC-calibrated average [Ca^{2+}] time course in the two regions of interests depicted in G, as indicated. Note a similar magnitude of near-rest [Ca^{2+}] fluctuations

in both branches confirming a similar dynamic range of the two recordings. Grey shade, Ca^{2+} rise interval in the *Active* branch.

(I) Fluorescence (NTC) decay time course for OGB-1 FLIM in the *Active* branch recording trace (shown in G-H) sampled outside Ca^{2+} rise interval (Resting, black), inside the interval (Rise, red), and throughout the recording (Overall, magenta). Including the active [Ca^{2+}] rise event appears to have little effect on the overall readout value.

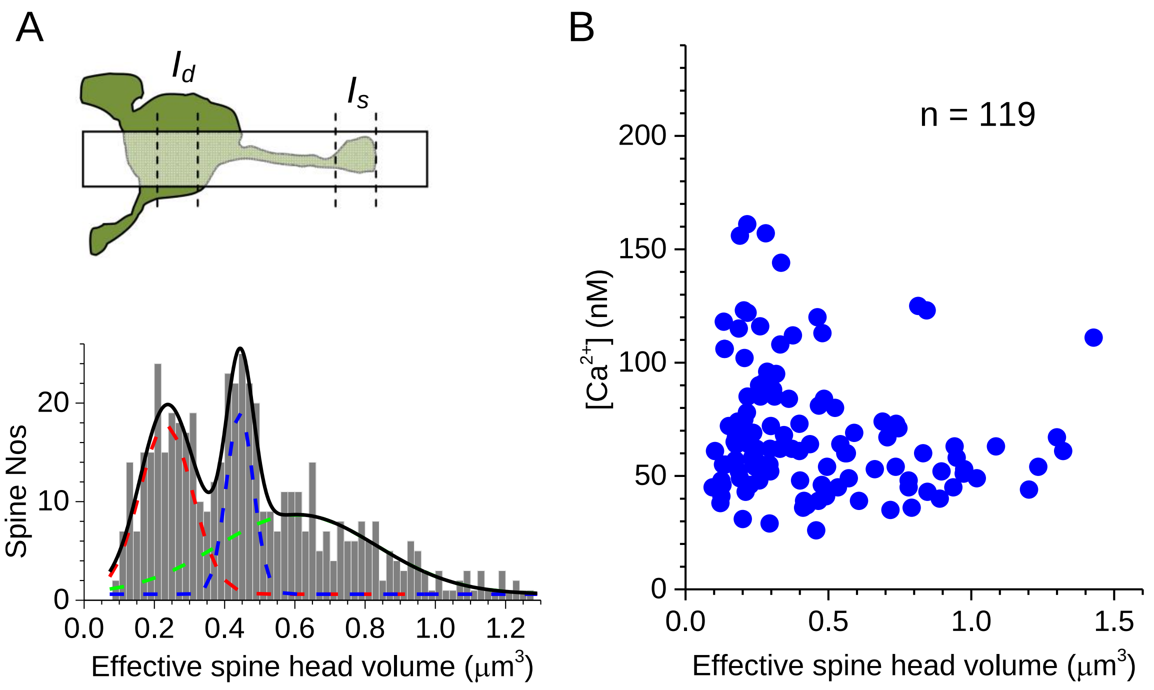

Figure S4 (Related to Fig. 4). The relationship between the size of dendritic spine heads and local resting [Ca^{2+}].

(A) Upper panel, diagram (vertical section of the dendritic fragment) depicting the spine head volume measurement which involves the ratio between the spine head fluorescence intensity (I_s) and that of the dendritic shaft in the same focal plane (I_d); white rectangle illustrates the characteristic focal two-photon excitation width: the volume scale was validated and adjusted *post hoc* using the average spine head size measurement (median width 519 nm) recently obtained in a spine population form the same hippocampal area using super-resolution (STED) microscopy (Tonnesen et al., 2014). Histogram, frequency distribution of spine head volumes (n = 536) suggests two or three spine subgroups with respect to head size. Dotted lines, three Gaussian distributions; solid line, the sum (histogram best fit).

(B) In proximal (first order) apical dendrites of CA1 pyramidal cells the spine head volume is not correlated with the intra-head [Ca^{2+}] measured with OGB-1 FLIM readout. Dots, individual dendritic spine recordings.

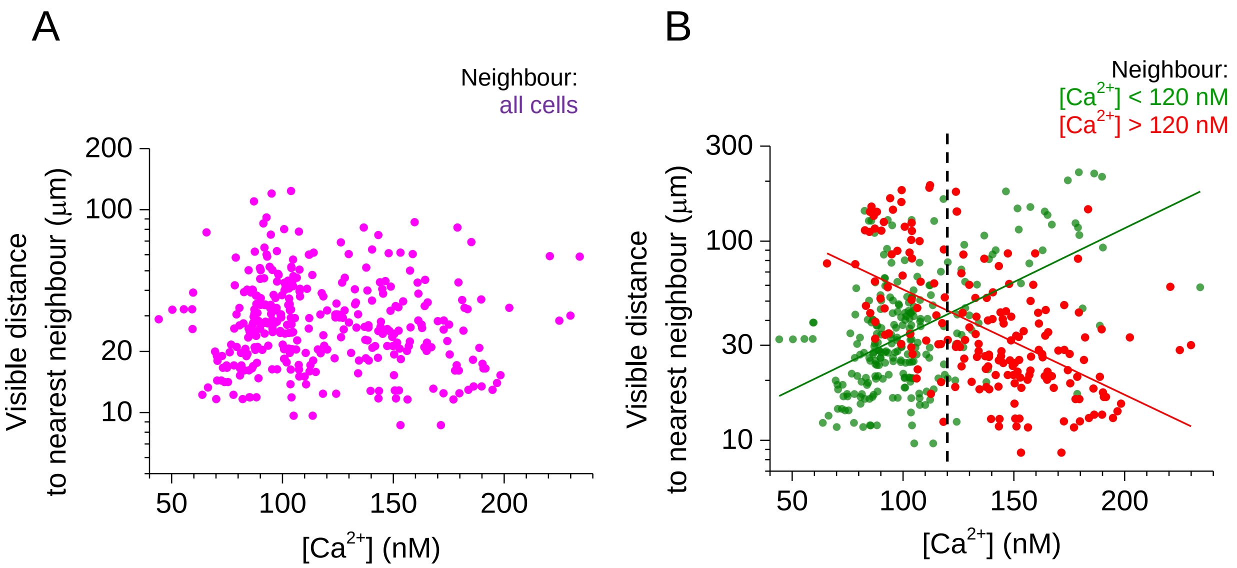

Figure S5 (Related to Fig. 5). Nearest-neighbour distances and somatic $[Ca^{2+}]$ in cortex astroglia *in vivo*.

(A) Distance to the nearest neighbor from individual, randomly sampled astrocytes plotted against $[Ca^{2+}]$ in the sampled astrocyte (n = 343 cells).

(B) Distance to the astrocyte's nearest neighbor plotted against astrocyte $[Ca^{2+}]$, with the nearest-neighbor astrocytes marked in accordance with one of their two somatic $[Ca^{2+}]$ sub-group (see Fig. 5E), as indicated: green, $[Ca^{2+}]$ < 120 nM (n = 301); red, $[Ca^{2+}]$ > 120 nM (n = 327), with the 120 nM boundary depicted by vertical dotted line. The plot indicates that the distance from any lower-$[Ca^{2+}]$ cell (green) to its nearest neighbor increases with higher $[Ca^{2+}]$ in that neighbor (green line, linear regression at $F = 88.7$, $p < 0.001$); conversely, the distance from any higher-$[Ca^{2+}]$ cell (red) to its nearest neighbor increase with lower $[Ca^{2+}]$ in that neighbor (red line, linear regression at $F = 81.8$, $p < 0.001$). In other words, astrocytes from the same $[Ca^{2+}]$ group tend to occur substantially closer to one another compared to those sampled from the two different groups.
